# Carbazole-Based Ester Derivatives as Potential α-Glucosidase Inhibitors; Synthesis, Biological Evaluation, and Molecular Docking Studies

**DOI:** 10.3390/molecules31122113

**Published:** 2026-06-16

**Authors:** Leyla Kaya, Mehmet F. Saglam, Rabia Sarıbas, Murat Bingul, Alev Arslantürk Bingül, Mahmut Yıldız, Hasan Sahin, Sadık Metin Ceyhan, Ustun Utkan Acar, Hakan Kandemir, Ibrahim F. Sengul

**Affiliations:** 1 Department of Pharmaceutical Toxicology, Faculty of Pharmacy, Zonguldak Bulent Ecevit University, Zonguldak 67600, Türkiye; 2 Department of Chemistry, Faculty of Science, Gebze Technical University, Kocaeli 41400, Türkiye; 3 Department of Basic Pharmaceutical Sciences, Faculty of Pharmacy, Dicle University, Diyarbakır 21280, Türkiye; 4 Department of Chemistry, Institute of Natural and Applied Sciences, Dicle University, Diyarbakır 21280, Türkiye; 5 Department of Pharmacognosy, Faculty of Pharmacy, Dicle University, Diyarbakır 21280, Türkiye; 6 Central Laboratory, Kocaeli University, Kocaeli 41001, Türkiye; 7 Department of Chemistry, Faculty of Art and Science, Tekirdag Namık Kemal University, Tekirdag 59030, Türkiye

**Keywords:** carbazole, ester, α-amylase, α-glucosidase, ADME, molecular modelling

## Abstract

In this study, a new range of carbazole linked mono- and bis-ester derivatives were successfully synthesized and evaluated for their *in vitro* anti-diabetic activity through α-amylase and α-glucosidase inhibition. The synthetic approach for the preparation of mono-esters **5a**–**j** and bis-esters **7a**–**j** was achieved by the reaction of carbazole-3-methanol **4** and carbazole-3,6-dimethanol **6** with a variety of acyl chlorides. The targeted compounds displayed generally weak α-amylase inhibition but significant and selective inhibition against α-glucosidase. Among them, **7a**, **7g**, and **7j** exhibited dual inhibitory activity, while **7f** was selective for α-glucosidase, and **7d** and **5g** for α-amylase. Notably, **7g** was the most potent compound (IC_50_ = 22.79 µM), surpassing Acarbose. In addition, molecular docking studies of the synthesized compounds were carried out to investigate their interactions with the α-glucosidase enzyme. Overall, targeted carbazole-based ester derivatives show promising results as selective anti-diabetic agents.

## 1. Introduction

Diabetes mellitus (DM) is a widespread, well-known, and escalating chronic group of metabolic conditions, characterized by sustained hyperglycemia resulting from inadequate insulin secretion, impaired insulin responsiveness, or the coexistence of these mechanisms [1,2]. Hyperglycemia in diabetes mellitus drives microvascular complications (retinopathy, neuropathy, nephropathy), increases risks of various malignancies, and contributes to cardiovascular and peripheral vascular disorders, thereby substantially elevating morbidity and mortality [3,4]. The global burden of diabetes is rising at an alarming rate; the International Diabetes Federation (IDF) estimated that approximately 589 million adults were living with diabetes in 2024, with projections anticipating a rise to 853 million by 2050 [5]. Diabetes mellitus is commonly classified as either type I (T1DM), type II (T2DM), or gestational DM. T2DM is the non-insulin-dependent form associated with progressive β-cell dysfunction and represents the vast majority of cases worldwide [6,7]. Targeting the suppression of carbohydrate-hydrolyzing enzymes, such as *α*-amylases and *α*-glucosidases, is a frontline intervention to mitigate postprandial hyperglycemia in diabetic patients [8,9]. *α*-Glucosidase (EC3.2.1.20) functions as a key digestive hydrolase, mediating the enzymatic hydrolysis of terminal 1,4-*α*-glycosidic bonds and releasing monosaccharide units from complex carbohydrates, facilitating glucose release and absorption [10]. *α*-Glucosidase inhibitors have been clinically efficacious as antihyperglycemic agents, as they effectively lower postprandial glycemia by postponing intestinal glucose release [11]. In parallel with *α*-glucosidase, pancreatic *α*-amylase (EC 3.2.1.1) functions as a critical enzyme in carbohydrate mechanism by converting long-chain carbohydrates into glucose, making it a complementary target for therapeutic intervention in T2DM [12,13]. Drugs such as Acarbose, miglitol, and voglibose are administered to attenuate enzymatic breakdown of carbohydrates and mitigate hyperglycemic responses. However, treatment with these drugs is often accompanied by side effects, including diarrhea, abdominal discomfort, and meteorism, resulting from the over-suppression of *α*-amylase and resultant bacterial fermentation of undigested carbohydrates in the colon [14,15]. Consequently, the pursuit of novel and more efficacious *α*-glucosidase and *α*-amylase inhibitors has become an area of expanding scientific interest.

Over the last few decades, considerable attention has been focused on design and synthesis of new heterocyclic compounds as potential α-glucosidase and α-amylase inhibitors. Among these heterocycles, the carbazole unit has emerged as one of the most promising structural motifs due to their versatile chemical and pharmacological properties. The carbazole core provides a rigid, planar, and π-rich pharmacophoric scaffold that can facilitate π–π stacking and hydrophobic interactions within the active sites of carbohydrate-hydrolyzing enzymes. In addition, the presence of a nitrogen atom within the heterocyclic framework may contribute to hydrogen bonding interactions with key amino acid residues in the catalytic pocket [16,17]. The ester functional group, one of the basic functional groups, is also quite important in medicinal chemistry. It is frequently used in the synthesis of prodrugs, which are compounds containing an ester group that are synthesized in a biologically inactive form and converted into a different functional group in the body, resulting in active drug structures [18]. To date, numerous carbazole derivatives have been investigated for their potential in treatment of diabetes [19]. For example, Ucar et al. reported the synthesis of a series of furocarbazole derivatives exhibiting significant α-glucosidase inhibitory activity, outperforming the standard drug Acarbose [16]. In another study, a range of carbazole–thiadiazole hybrids have been synthesized and investigated for their potent α-amylase and α-glucosidase inhibitory activities, highlighting the potential of carbazole derivatives as promising anti-diabetic agents [20]. Building on these findings, in the present study, we synthesized a series of ester derivatives based on carbazole scaffolds and evaluated their α-glucosidase and α-amylase inhibitory activities to explore their potential as anti-diabetic agents. To further modulate the physicochemical and biological properties of this scaffold, ester functionalities were introduced at different substitution patterns. Mono-ester derivatives were designed to maintain partial polarity and improve potential binding orientation, whereas bis-ester derivatives were synthesized to increase molecular flexibility, hydrogen bonding capacity, and hydrophobic surface area. This scaffold diversification strategy was aimed at exploring the influence of substitution degree on α-glucosidase and α-amylase inhibitory activities in a systematic manner.

## 2. Results and Discussion

### 2.1. Synthesis and Characterization

**Scheme 1:**
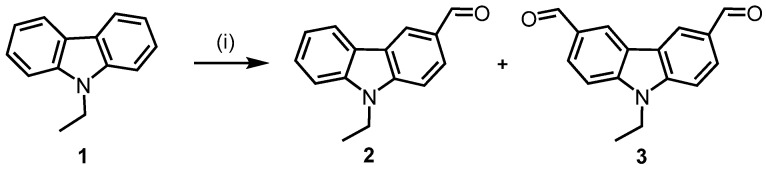
Synthesis of carbazole-3-carbaldehyde **2** and carbazole-3,6-dicarbaldehyde **3**. Reagents and conditions: (i) DMF, POCl_3_, 100 °C.

It is well established that the C3 and C6 positions of the carbazole ring are the most reactive sites toward electrophilic substitution, enabling efficient functionalization and covalent linkage to other molecular frameworks [21]. The synthesis of carbazole-linked mono- and bis-esters was initiated by the formylation of *N*-ethylcarbazole **1** using POCl_3_/DMF under Vilsmeier–Haack reaction conditions, producing carbazole-3-carbaldehyde **2** and carbazole-3,6-dicarbaldehyde **3**, which were separated by flash column chromatography [22] (Scheme 1).

**Scheme 2:**
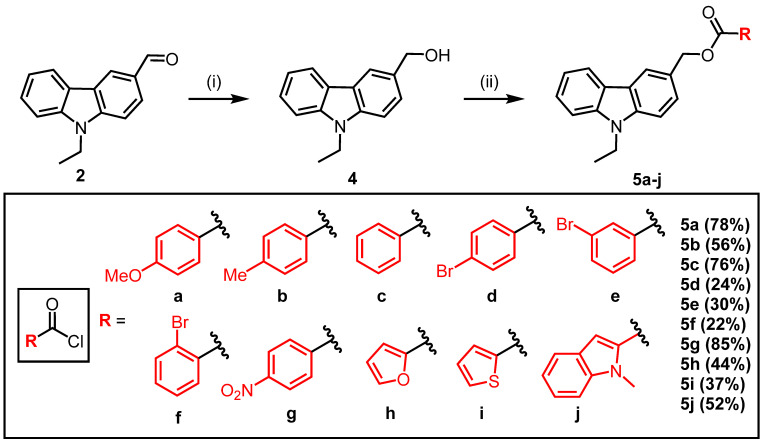
Carbazole-3-carbaldehyde **2** reduction to corresponding alcohol **4** and synthesis of carbazole-based mono-ester derivatives **5a**–**j**. Reagents and conditions: (i) NaBH_4_, EtOH:THF (9:1), 25 °C, 1 h; (ii) acyl chlorides, DCM, Et_3_N, 0 °C to 25 °C.

**Scheme 3:**
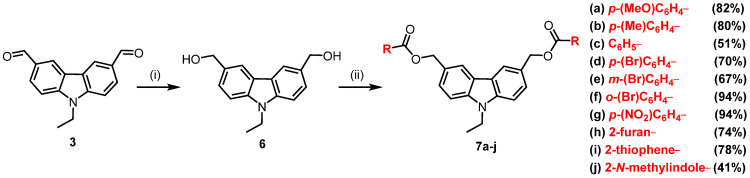
Carbazole-3,6-dicarbaldehyde **3** reduction to corresponding alcohol **6** and synthesis of carbazole-based bis-ester derivatives (**7a**–**j**). Reagents and conditions: (i) NaBH_4_, EtOH:THF (2:1), 25 °C, 1 h; (ii) acyl chlorides, DCM, Et_3_N, 0 °C to 25 °C.

Subsequently, the synthesized mono formyl *N*-ethylcarbazoles **2** bearing aldehyde functional group was reduced with NaBH_4_ in ethanol and tetrahydrofuran to produce the corresponding alcohol derivative, which serve as key intermediates for the esterification reaction, yielding carbazole-3-methanol **4** (Scheme 2) and carbazole-3,6-dimethanol **6** (Scheme 3) [23,24].

**Figure 1:**
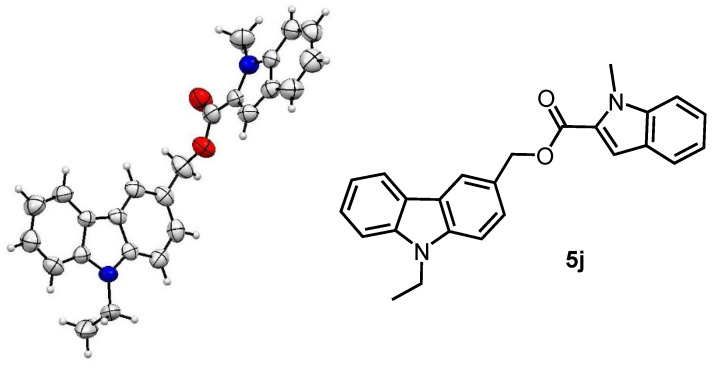
The ORTEP diagram and chemical structure of the compound **5j** drawn at the 50% probability level show that the aromatic core is essentially planar, with well-defined thermal ellipsoids. Hydrogen atoms are shown as spheres of arbitrary radius.

Following the preparation of the carbazole-3-methanol **4** and carbazole-3,6-dimethanol **6**, a monomeric model was first employed for the synthesis of mono-esters **5a-j** prior to extending the approach to bis-esters **7a**–**j** (Scheme 2). Initially, carbazole-3-methanol **4** was subjected to esterification with *p*-methoxybenzoyl chloride in dry dichloromethane in the presence of triethylamine at 0 °C under an argon atmosphere. The reaction mixture was subsequently allowed to warm to room temperature and stirred overnight to ensure completion. After standard aqueous work-up and extraction, the crude product was purified by silica gel flash column chromatography (hexane/ethyl acetate, 75:25) to produce the corresponding *p*-methoxy-substituted carbazole ester **5a** with a 78% yield as a light-yellow oil. Similarly, treatment of carbazole-3-methanol **4** with other benzoyl chlorides and heterocyclic acyl chlorides under the same reaction conditions produced the corresponding mono-ester derivatives **5b**–**j** in yields ranging from 22% to 85%. Compounds **5a**–**j** were new, and their structures were confirmed by spectroscopic analysis. The ^1^H and ^13^C NMR data of compound **5a** were found to be representative for the entire series of **5a**–**j**. In the ^1^H NMR spectrum, a singlet at 5.52 ppm was attributed to the -OC*H*_2_- protons. The methylene protons adjacent to the nitrogen atom (-NC*H*_2_-) appeared as a quartet at 4.48 ppm, while the terminal ethyl C*H*_3_ group resonated as a triplet at 1.44 ppm. In addition, the methoxy substituent (-OC*H*_3_) on the benzene ring exhibited a singlet at 3.87 ppm. In the ^13^C NMR spectrum of the compound **5a**, the carbonyl carbon signal was observed at 166.51 ppm. The -O*C*H_2_- carbon resonated at 67.52 ppm, while the methoxy carbon (-O*C*H_3_) appeared at 55.49 ppm. Furthermore, the carbons corresponding to the -N*C*H_2_- and terminal -*C*H_3_ groups were detected at 37.71 and 13.89 ppm, respectively. Crystallization studies produced suitable single crystals of the indole ester derivative **5j**, whose structure was subsequently confirmed by single-crystal X-ray crystallography (Figure 1). The molecular structure of **5j** crystallized in the orthorhombic Pbca space group. The bond lengths within the aromatic rings (1.36–1.40 Å) confirmed the presence of delocalized π-electron systems. The N1 atom exhibited sp^2^ hybridization and participated in conjugation with adjacent aromatic rings. The C=O bond length (1.210 Å) clearly indicates a carbonyl functionality. Torsion angle analysis revealed that while the core aromatic system was largely planar, substituent groups showed deviations from planarity, indicating conformational flexibility. The relatively high Rint value suggests moderate crystal quality, although the final refinement parameters were within acceptable limits.

Following the successful application of this esterification method for the synthesis of monomeric carbazole-linked esters **5a**–**j**, the preparation of related bis-ester systems was subsequently investigated. In this context, carbazole-3,6-dimethanol **6** was subjected to reaction with benzyl chlorides bearing electron-donating and electron-withdrawing groups, as well as heterocyclic acyl chlorides, in the presence of dry DCM and triethylamine. This transformation produced the desired carbazole-based bis-ester derivatives **7a**–**j**, with yields ranging from 41 to 94% (Scheme 3). The structures of bis-esters **7a**–**j** were supported by ^1^H NMR and ^13^C NMR spectroscopic data. The ^1^H NMR spectrum of compound **7a** in CDCl_3_, as a representative example of the carbazole-linked bis-esters **7a**–**j**, displayed a singlet at 5.52 ppm corresponding to the benzylic protons (-OC*H*_2_-). The carbazole -NC*H*_2_- and -C*H*_3_ protons appeared as a quartet and a triplet at 4.36 ppm and 1.43 ppm, respectively. The methoxy protons (-OC*H*_3_) appeared as a singlet at 3.84 ppm. The ^13^C NMR spectrum showed signals at 13.86 and 37.77 ppm, corresponding to the -*C*H_3_ and -N*C*H_2_- carbons, respectively. In addition, the spectrum displayed a carbonyl carbon (-*C*OO-) signal at 166.45 ppm and a methoxy carbon (-O*C*H_3_) resonance at 55.44 ppm.

### 2.2. Biological Studies

#### 2.2.1. Determination of Anti-Diabetes Activity

The anti-diabetic potential of the synthesized compounds was evaluated by determining their half-maximal inhibitory concentrations (IC_50_) against α-amylase and α-glucosidase enzymes. These values were subsequently compared with those of Acarbose, a widely used reference inhibitor, to assess the relative enzyme inhibitory efficacy of the tested compounds. In this study, carbazole-based mono- and bis-ester derivatives (**5a**–**j** and **7a**–**j** series) were examined across eight different concentration levels, ranging from 6.75 to 800 μM for both α-glucosidase and α-amylase. The resulting IC_50_ values are summarized in Table 1. Compounds exhibiting lower IC_50_ values were considered to possess stronger inhibitory activity and, therefore, greater potential as anti-diabetic agents. As expected, the standard drug Acarbose demonstrated potent inhibitory activity, with IC_50_ values of 1033.8 μM for α-glucosidase and 0.1 μM for α-amylase (Table 1).

Among the evaluated compounds, derivatives belonging to the bis-ester carbazole series exhibited comparatively stronger inhibitory activity against α-glucosidase than their mono-ester counterparts. In particular, compounds **7a**, **7g**, **7f**, and **7j** demonstrated more favourable IC_50_ values relative to the remaining derivatives. Notably, compound **7g** showed the highest α-glucosidase inhibitory potency, with an IC_50_ value of 22.79 μM, markedly surpassing the reference inhibitor Acarbose (IC_50_ = 1033.8 μM). Compound **7a** also displayed moderate activity (IC_50_ = 54.06 μM), suggesting that specific substituents on the carbazole scaffold may enhance interactions within the enzyme active site.

In contrast, the majority of the tested compounds (**7b**–**e**, **7h**–**i**, and **5a**–**j** derivatives) exhibited weak or negligible inhibition (IC_50_ > 800 μM), indicating that their structural features are not optimal for effective α-glucosidase inhibition. However, compound **5g**, representing a mono-aryl ester carbazole derivative, showed weak yet selective inhibition toward α-amylase (IC_50_ = 635.3 μM), pointing to possible enzyme-specific binding preferences. A similar trend was observed for selected bis-ester derivatives, including **7a**, **7d**, **7g**, and **7j**, which demonstrated measurable activity against α-amylase. Among these, **7g** again emerged as the most active compound, with an IC_50_ value of 48.08 μM.

Regarding α-amylase inhibition, although **7g** exhibited notable activity, it remained less potent than Acarbose (IC_50_ = 0.1 μM). Compound **7j** displayed moderate dual inhibitory activity, with IC_50_ values of 475.1 μM for α-glucosidase and 157.5 μM for α-amylase, indicating a broader yet less pronounced inhibitory profile. On the other hand, compound **7f** showed relatively higher selectivity toward α-glucosidase, with an IC_50_ value of 614.0 μM, but negligible activity against α-amylase.

Overall, these findings identify **7g** as the most promising candidate, exhibiting potent dual inhibitory activity, especially against α-glucosidase. Its superior performance compared to Acarbose suggests that its structural features may facilitate stronger binding interactions within the enzyme active site. These results warrant further investigation through molecular docking and kinetic studies to elucidate structure–activity relationships and to support the rational design of more effective anti-diabetic agents.

The structure–activity relationship analysis revealed that the number of aryl ester substituents on the carbazole scaffold play a critical role in modulating enzyme inhibitory activity. In general, bis-ester carbazole derivatives **7a**–**j** demonstrated superior α-glucosidase inhibitory activity compared to the mono-ester counterparts **5a**–**j**, suggesting that increased aromatic substitution enhances interactions within the enzyme active site, likely through improved hydrophobic contacts and π–π stacking interactions.

Among the **7a**–**j** series, compound **7g**, bearing electron-withdrawing nitro substituents, exhibited the highest activity against both α-glucosidase and α-amylase. This observation indicates that strong electron-withdrawing groups may enhance binding affinity, possibly by increasing electrophilic character and facilitating stronger hydrogen bonding or dipole–dipole interactions with catalytic residues. Similarly, **7a**, which also contains polar functional groups, showed moderate activity, further supporting the positive contribution of polar/electron-withdrawing substituents.

In contrast, compounds such as **7b**–**e** and **7h**–**i**, which lack strongly activating substituents or possess bulkier/non-polar groups, exhibited significantly reduced activity (IC_50_ > 800 μM). This suggests that steric hindrance and insufficient electronic activation negatively affect binding efficiency. For example, halogenated derivatives such as **7d** (*p*-bromo-substituted) showed weak activity, indicating that while halogens may contribute to lipophilicity, excessive steric bulk may limit proper accommodation within the enzyme active pocket.

The mono-ester carbazoles **5a**–**j** generally displayed poor inhibitory activity, highlighting the importance of molecular extension and increased aromatic surface area for effective enzyme interaction. However, **5g** showed weak but selective α-amylase inhibition, implying that simpler mono-ester frameworks may still retain limited enzyme-specific affinity, potentially due to better fit within narrower binding regions.

Compound **7j**, incorporating indole fragments with the additional nitrogen atoms, demonstrated moderate dual inhibition, suggesting that nitrogen atoms may contribute to binding via hydrogen bonding or coordination interactions, although not sufficient to achieve high potency.

To provide a more comprehensive understanding of the observed inhibitory activities, selected physicochemical descriptors including molecular weight (MW), calculated lipophilicity (cLogP (XLOGP3 and Concensus Log P_o/w_)), and topological polar surface area (TPSA) were calculated using the SwissADME web platform. The compound **7a** exhibited a molecular weight of 523.58 g/mol, cLogP values of 6.27 and 5.21, and TPSA of 75.99 Å. The presence of para-methoxy substituents provides a moderate electron-donating character through resonance while maintaining an appropriate balance between lipophilicity and polarity. The relatively high lipophilicity may favour hydrophobic contacts within the α-glucosidase binding pocket, whereas the methoxy oxygen atoms contribute additional hydrogen bond acceptor sites. Such a balance is generally advantageous for stabilizing ligand–enzyme interactions and may explain the favourable activity observed for methoxy-substituted analogues. In contrast, the bis(4-nitrobenzoyl) derivative (compound **7g**) displayed a higher molecular weight (553.52 g/mol) and a markedly increased TPSA (149.17 Å^2^), all while maintaining comparable cLogP values of 5.99 and 3.72. The nitro group is a strong electron-withdrawing substituent that decreases electron density on the aromatic ring and can influence the electrostatic complementarity of the ligand toward amino acid residues within the active site. Moreover, the nitro groups increase the number of hydrogen bond acceptors and substantially enhance molecular polarity. The fused heteroaromatic bis-ester derivative **7j** possesses the highest molecular weight (569.65 g/mol) and lipophilicity (cLogP = 7.16 and 5.66) among the representative compounds, while exhibiting the lowest TPSA (67.39 Å^2^). The extended aromatic framework significantly increases hydrophobic surface area and π-electron density, potentially promoting π–π stacking and van der Waals interactions with aromatic residues located in the binding pocket. However, the increased steric bulk associated with the fused bicyclic substituents may restrict conformational flexibility and hinder optimal fitting within the active site. Consequently, the biological activity of this derivative is expected to depend on whether the enzyme cavity can accommodate the larger aromatic system without significant steric clashes. Compared with the bis-substituted ester derivatives **7a** and **7g**, the mono-substituted ester compound **5g** exhibited a lower molecular weight (374.39 g/mol) and a moderate TPSA value of 75.05 Å^2^ while maintaining comparable lipophilicity cLogP (4.81 and 3.55). The introduction of a second aryl ester moiety did not substantially increase the overall lipophilic character of the molecules while and only increased the molecular weight of the compound, suggesting an increase in molecular size and structural complexity, which may influence their binding interactions within the enzyme active site.

Comparison of these representative compounds indicates that inhibitory activity is governed by a combination of electronic, lipophilic, and steric factors rather than by substituent identity alone. Electron-withdrawing groups such as nitro substituents may strengthen enzyme recognition through favourable electronic interactions, whereas electron-donating methoxy groups provide a balanced combination of lipophilicity and hydrogen bonding capability. Likewise, the superior activity often observed for bis-ester derivatives relative to mono-substituted analogues may arise from their larger hydrophobic surface area and increased capacity to engage multiple regions of the binding pocket simultaneously. Overall, the data suggest that optimal α-glucosidase inhibition requires a balance between molecular size, lipophilicity, polarity, and electronic characteristics, with excessive steric bulk or polarity potentially limiting activity despite otherwise favourable structural features.

#### 2.2.2. Molecular Docking Studies

All molecular docking figures were re-generated using high-resolution visualization settings to improve the representation of the enzyme active sites and protein–ligand interactions. Hydrogen bonds, hydrophobic contacts, and other interaction types were distinguished using consistent colour coding, while catalytic and key binding site residues were explicitly labelled. Additional three-dimensional overview panels illustrating the ligand orientation within the catalytic pocket and the spatial arrangement of important active site residues were included for both α-amylase and α-glucosidase to facilitate structural interpretation and comparison of the docking results.

The compounds with the highest inhibition values obtained from biological assays have been evaluated for molecular docking studies. The interactions between the best candidates and the amino acid residues on the catalytic side of the enzymes are given below.

**Figure 2:**
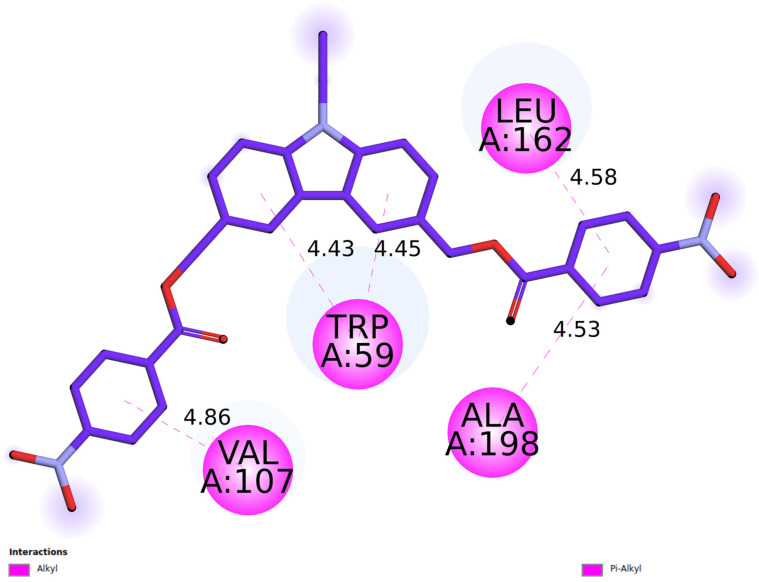
2D representation of **7g** and α-amylase enzyme binding site residue interactions.

**Figure 3:**
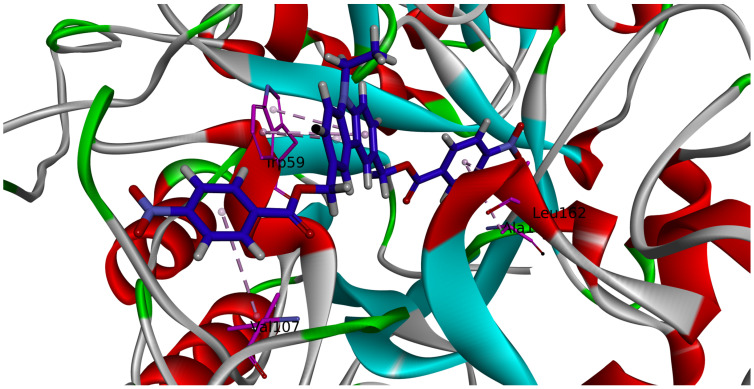
3D representation of **7g** and α-amylase enzyme binding site residue interactions.

The molecular docking analysis of compound **7g** within the active site of the α-amylase enzyme revealed a stable binding conformation predominantly stabilized by hydrophobic interactions (Figure 2 and Figure 3). As illustrated in the binding pose, **7g** is well accommodated within the catalytic pocket, forming key π-alkyl and alkyl interactions with residues TRP59, VAL107, LEU162, and ALA198. The aromatic scaffold of the compound establishes favourable π–alkyl interactions with TRP59, which is known to play an important role in substrate recognition and stabilization within the enzyme active site. Additionally, the hydrophobic side chains of VAL107 and LEU162 further contribute to ligand stabilization through van der Waals and alkyl contacts, enhancing the overall binding affinity. The presence of these non-polar interactions suggests that the bulky bis-ester carbazole framework of **7g** is well-suited for occupying the hydrophobic cavity of α-amylase. Although no strong hydrogen bonding interactions are observed, the cumulative effect of these hydrophobic contacts appears sufficient to anchor the ligand effectively, which is consistent with its experimentally observed potent inhibitory activity.

**Figure 4:**
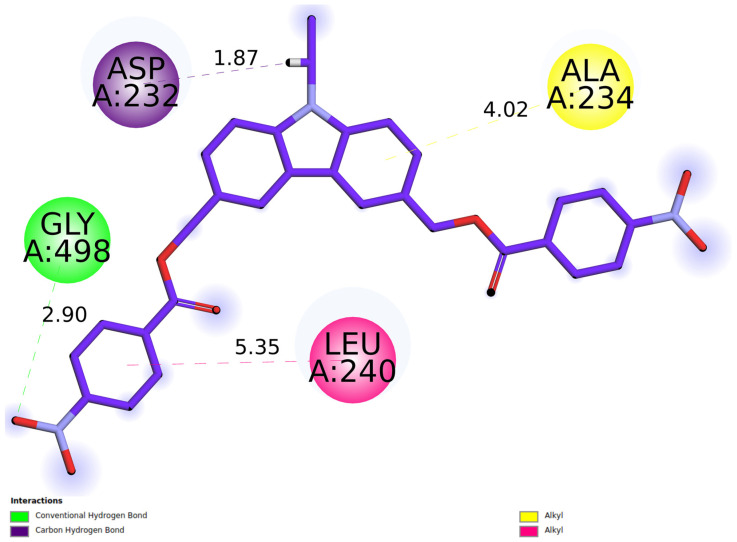
2D representation of **7g** and α-glucosidase enzyme binding site residue interactions.

**Figure 5:**
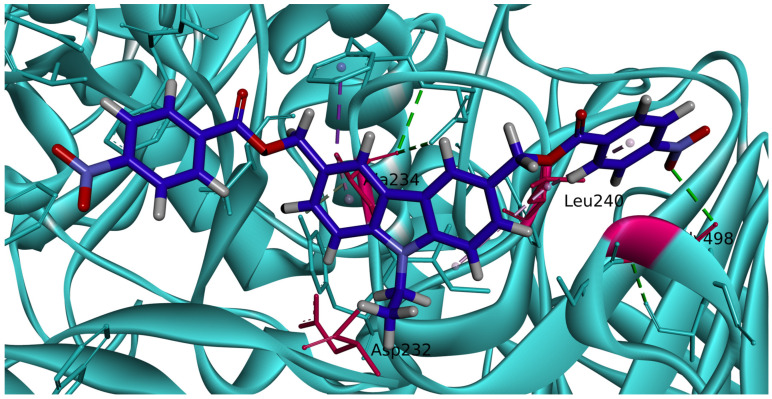
3D representation of **7g** and α-glucosidase enzyme binding site residue interactions.

In the case of α-glucosidase enzyme, the key interaction was detected as strong conventional hydrogen bonding formed between the GLY498 residue and nitro substitution located on the aryl ester fragment (Figure 4 and Figure 5). The presence of ASP232 for the formation of carbon–hydrogen bond interaction with the ligand’s aryl ring was also found to be a critical interaction for the ligand’s best placement in the α-glucosidase enzyme pocket. The hydrophobic interactions were built as alkyl contacts of LEU240 and ALA234 residues. The stabilization of the aromatic framework of the ligand within the enzyme’s hydrophobic pocket obtained by these interactions cooperated with the benzene ring on the carbazole moiety.

**Figure 6:**
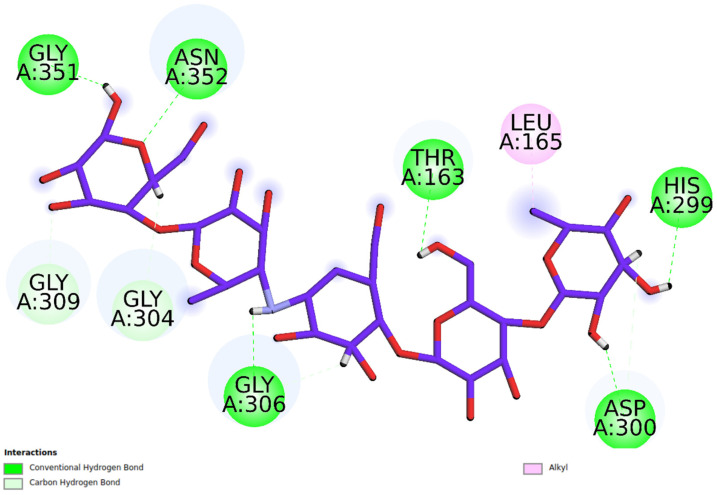
2D representation of Acarbose and α-amylase enzyme binding site residue interactions.

Acarbose exhibits its well-established inhibitory potency against amylase primarily due to its structural mimicry of natural oligosaccharide substrates and its ability to form an extensive and highly cooperative hydrogen bonding network within the active site (Figure 6). The interaction profile of the ligand within the active site of the amylase enzyme reveals a dense hydrogen bonding network supported by both conventional and carbon–hydrogen interactions, which collectively stabilize the ligand–enzyme complex. Key residues such as GLY351 and ASN352 appear to play a critical role in anchoring the ligand through conventional hydrogen bonds, suggesting their importance in substrate recognition and binding orientation. Additional stabilization is provided by GLY304, GLY306, and GLY309, which contribute multiple carbon–hydrogen interactions, reinforcing the ligand’s positioning within the binding pocket. The involvement of THR163 further enhances binding affinity via hydrogen bonding, likely facilitating proper alignment of the ligand for optimal interaction with catalytic residues. Notably, HIS299 and ASP300, residues often implicated in catalytic activity, also participate in hydrogen bonding, indicating that the ligand is well-positioned within the catalytic region of the enzyme. Furthermore, the presence of an alkyl interaction with LEU165 suggests a hydrophobic contribution to binding stability, complementing the predominantly polar interaction network. Overall, this combination of hydrogen bonding and hydrophobic contacts highlights a strong and specific binding mode, which may explain the observed inhibitory potential of the compound against the amylase enzyme.

**Figure 7:**
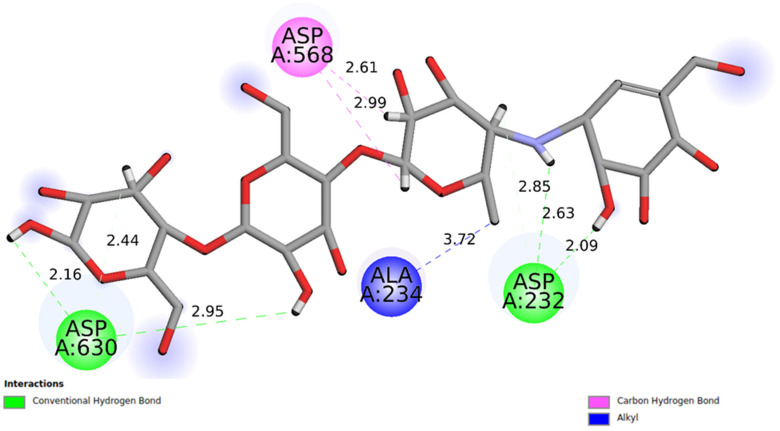
2D representation of Acarbose and α-glucosidase enzyme binding site residue interactions.

The binding mode of Acarbose within the α-glucosidase active site further reinforces its role as a highly potent anti-diabetic inhibitor by demonstrating a dense and catalytically relevant interaction network (Figure 7). The ligand establishes multiple strong conventional hydrogen bonds with key acidic residues, particularly Asp232 and Asp630, with short interaction distances, indicating highly stable contacts. These residues are typically involved in the catalytic mechanism of α-glucosidase, functioning as proton donors/acceptors during glycosidic bond cleavage. The additional interaction with ASP568 through carbon–hydrogen bonding further stabilizes the ligand within the catalytic pocket, suggesting that Acarbose effectively spans a large portion of the active site.

Importantly, the extensive hydroxyl functionality of Acarbose allows it to closely mimic the natural carbohydrate substrates of α-glucosidase, enabling the formation of a cooperative hydrogen bonding network that locks the enzyme in an inactive conformation. The interaction with ALA234 via a weaker alkyl contact contributes to fine-tuning the ligand orientation, ensuring optimal positioning of polar groups toward catalytically important residues. The overall binding pattern indicates that Acarbose not only occupies the substrate-binding region but also directly engages residues essential for catalysis, thereby acting as a competitive inhibitor.

This strong multi-point attachment, dominated by short-distance hydrogen bonds with catalytic aspartate residues, is a key reason why Acarbose exhibits high inhibitory efficiency against α-glucosidase. By stabilizing a non-reactive enzyme–inhibitor complex and preventing substrate access, it effectively delays carbohydrate hydrolysis and glucose absorption, which underlies its clinical utility in the management of postprandial hyperglycemia in diabetes.

To provide a more comprehensive evaluation of the molecular docking results and to support the observed structure–activity relationships, representative compounds spanning different activity levels were additionally subjected to docking analysis. The calculated docking scores for highly active, moderately active, and less active analogues are summarized in Table 2. Comparison of the docking results with the experimental α-amylase and α-glucosidase inhibition data revealed a general correlation between favourable binding energies and enhanced inhibitory activity. Although compound **7g** exhibited the strongest binding affinity and was therefore selected for detailed interaction analysis, the inclusion of additional analogues demonstrates that the docking methodology captures the overall activity trend within the compound series and provides further support for the proposed binding mode and SAR interpretations.

The docking results reveal a clear difference in binding affinities between compound **7g** and Acarbose toward both α-amylase and α-glucosidase enzymes. Acarbose exhibited significantly more favourable docking scores (−70.03 kcal/mol for α-amylase and −62.27 kcal/mol for α-glucosidase) compared to **7g** (−46.27 and −25.60 kcal/mol). This substantial energy difference suggests that Acarbose forms a much more stable enzyme–ligand complex in both targets. The superior binding affinity of Acarbose can be rationalized by its highly hydroxylated pseudo-oligosaccharide structure, which enables extensive hydrogen bonding with catalytic residues and strong mimicry of natural carbohydrate substrates. In contrast, although **7g** demonstrates moderate binding particularly toward α-amylase its comparatively less polar structure likely limits the number and strength of interactions within the active site, especially in α-glucosidase where the score is notably weaker. Although the compound **7j** demonstrated the most favourable docking performance, showing docking scores of −57.08 kcal/mol against α-amylase and −37.67 kcal/mol against α-glucosidase. The *in vitro* enzyme inhibition assays revealed superior anti-diabetic activity for **7g**. This apparent discrepancy highlights a well-recognized limitation of molecular docking, whereby docking scores represent only an estimation of the binding free energy based on a static protein structure and may not fully reflect the complex nature of enzyme inhibition under biological conditions. The relatively strong binding of **7j** suggests that its structural features may facilitate effective interactions within the catalytic pocket, potentially through hydrogen bonding, hydrophobic contacts, and π-related interactions with key active site residues. Compound **7a** displayed moderate binding affinity, similar to compound **7g**, toward both enzymes, yielding docking scores of −42.51 kcal/mol for α-amylase and −21.01 kcal/mol for α-glucosidase. Since compounds **5g** and **7f** showed activity toward only one enzyme, docking studies were conducted solely on their corresponding active enzyme targets. Compound **5g** showed moderate affinity for α-amylase (−36.37 kcal/mol) and compound **7f** demonstrated only weak binding toward α-glucosidase (−11.60 kcal/mol). Overall, these findings highlight Acarbose as a benchmark inhibitor with optimal interaction complementarity, while **7g**, despite lower binding energies, may still serve as a promising scaffold for further structural optimization aimed at enhancing hydrogen bonding capacity and improving inhibitory potency against carbohydrate-hydrolyzing enzymes.

#### 2.2.3. Evaluation of ADME Results

The pharmacokinetic profile of compound **7g** reveals several limitations that may influence its drug-likeness despite its promising biological activity (Table 3). The compound exhibits low gastrointestinal (GI) absorption, suggesting that its oral bioavailability may be limited, which could reduce its effectiveness when administered orally. Additionally, **7g** is predicted to be non-permeant to the blood–brain barrier (BBB), indicating that it is unlikely to exert central nervous system effects, which may be advantageous or disadvantageous depending on the therapeutic target. The bioavailability score of 0.17 further supports the notion of poor systemic availability.

In terms of cytochrome P450 enzyme interactions, **7g** does not inhibit CYP1A2, CYP2C19, CYP2D6, or CYP3A4, which is favourable as this reduces the risk of drug–drug interactions. However, it is predicted to inhibit CYP2C9, suggesting a potential for metabolic interference with drugs processed by this isoform. Overall, while **7g** demonstrates a relatively safe CYP inhibition profile, its poor absorption and low bioavailability highlight the need for structural optimization or formulation strategies to improve its pharmacokinetic properties.

## 3. Materials and Methods

### 3.1. Chemistry

Reactions were performed under a positive pressure of argon in oven- or flame-dried glassware, unless otherwise specified. All reagents and solvents were obtained from commercial sources and appropriately purified, if necessary. ^1^H and ^13^C NMR spectra were recorded at 298 K on a Varian 500 MHz NMR spectrometer (Palo Alto, Santa Clara, CA, USA). The deuterated solvent deutero chloroform (CDCl_3_) for NMR spectroscopy was obtained from Merck (Darmstadt, Germany). Residual chloroform (CHCl_3_ in CDCl_3_) (δ = 7.26 ppm) was used for ^1^H NMR spectra, and the central line of the chloroform (CDCl_3_) triplet (δ = 77.10 ppm) was used for ^13^C NMR spectra as an internal reference. The following abbreviations and combinations thereof are used to describe ^1^H NMR spectra multiplicities: s = singlet, d = doublet, t = triplet, q = quartet, m = multiplet, and br. = broad. IR spectra were recorded between 4000 and 650 cm^−1^ using a Perkin Elmer Spectrum 100 FT-IR spectrometer (PerkinElmer, Waltham, MA, USA). MALDI-TOF mass spectra were recorded on a Bruker Daltonics microTOF (Karlsruhe, Germany). Analytical TLC was performed with Merck silica gel plates, which were precoated with silica gel 60 F254 (0.2 mm) on aluminum sheets and visualized using UV fluorescence (λmax = 254 nm). Flash column chromatography employed Merck Kieselgel 60 (230–400 mesh) silica gel (Darmstadt, Germany).

All commercially available reagents and the standards used for the biological assays were purchased from Sigma-Aldrich (St. Louis, MO, USA). and carried out without further purification. In addition, α-amylase type VI-B (E.C. 3.2.1.1, from porcine pancreas, lyophilized powder), 2-chloro-4-nitrophenyl-α-D-maltotrioside (CNP-G3), sodium chloride, sodium azide, Acarbose, and DMSO were purchased from Sigma-Aldrich (St. Louis, MO, USA). All other chemicals were of analytical grade. Similarly, *p*-nitrophenol, α-D-glycopyranoside (PNPG), and α-glucosidase type I (EC 3.2.20, from *Saccharomyces cerevisiae*, lyophilized powder) were obtained from Sigma-Aldrich (St. Louis, MO, USA).

#### Synthesis of Mono- and Bis-Ester Carbazole Compounds

*General Procedure-1 (GP-1)—synthesis of mono-ester carbazole compounds* (**5a**–**j**): A reaction flask was charged with carbazole (1.0 mol equiv.) and dry DCM, then cooled to 0 °C under argon atmosphere. To this, dry Et_3_N (2.0 mol equiv.) was added via dropwise portion, then a solution of acyl chloride (1.5 mol equiv.) in dry DCM was added and stirred at 0 °C for 5 min. The reaction mixture was then warmed up to room temperature and stirred for 6–24 h. The resulting reaction mixture was then poured into water and extracted. The organic phase was separated, the inorganic phase was further extracted with DCM, all the organic phases were combined, and then washed with aqueous 1M HCl solution followed by *sat.* aqueous NaHCO_3_ solution. The organic phases were then combined, washed with brine, dried over Na_2_SO_4_, filtered, and concentrated under reduced pressure. Purification by silica gel flash column chromatography gave the target product **5a**–**j**.

*(9-Ethyl-9H-carbazol-3-yl)methyl 4-methoxybenzoate* (**5a**): The compound **5a** was prepared according to *GP-1* from carbazole (50.0 mg, 0.222 mmol, 1.0 mol equiv.), dry DCM (6 mL), dry Et_3_N (0.062 mL, 0.444 mmol, 2.0 mol equiv.), and *p*-methoxybenzoyl chloride (61.8 mg, 0.333 mmol, 1.5 mol equiv.) in 1 mL of dry DCM, and then stirred for 17 h. Purification by silica gel flash column chromatography (hexane:EtOAc 75:25) gave the target product **5a** (62 mg, 78% yield) as a light-yellow-coloured oil. R*_f_* 0.40 (in EtOAc:hexane 75:25); ^1^H NMR (500 MHz, CDCl_3_) *δ* 8.20 (s, 1H), 8.12 (d, *J* = 7.7 Hz, 1H), 8.05 (d, *J* = 8.2 Hz, 2H), 7.58 (d, *J* = 8.3 Hz, 1H), 7.51–7.45 (m, 1H), 7.42 (d, *J* = 8.3 Hz, 2H), 7.26–7.21 (m, 1H), 6.90 (d, *J* = 8.2 Hz, 2H), 5.52 (s, 2H), 4.38 (q, *J* = 7.2 Hz, 2H), 3.85 (s, 3H), 1.44 (t, *J* = 7.2 Hz, 3H) ppm; ^13^C NMR (125 MHz, CDCl_3_) *δ* 166.51 (COO), 163.41, 140.39, 139.94, 131.83, 126.79, 126.67, 125.93, 123.08, 122.96, 122.85, 121.27, 120.63, 119.06, 113.64, 108.64, 108.54, 67.52 (OCH_2_), 55.49 (OCH_3_), 37.71 (NCH_2_), 13.89 (CH_3_) ppm; FT-IR: υ_max_ 2932, 1707, 1605, 1511, 1491, 1471, 1327, 1253, 1232, 1166, 1096, 1027, 770 cm^−1^; MALDI-TOF (*m*/*z*) calc. for C_23_H_21_NO_3_: 359.425; found [M]^+^: 359.028.

*(9-Ethyl-9H-carbazol-3-yl)methyl 4-methylbenzoate* (**5b**): The compound **5b** was prepared according to *GP-1* from carbazole (50.0 mg, 0.222 mmol, 1.0 mol equiv.), dry DCM (7 mL), dry Et_3_N (0.062 mL, 0.444 mmol, 2.0 mol equiv.), and *p*-methylbenzoyl chloride (44 µL, 0.333 mmol, 1.5 mol equiv.) and stirred for 96 h. Purification by silica gel flash column chromatography (hexane:DCM 66:33) gave the target product **5b** (43 mg, 56% yield) as a light-yellow-coloured oil. R*_f_* 0.40 (in hexane:DCM 66:33); ^1^H NMR (500 MHz, CDCl_3_) *δ* 8.18 (d, *J* = 1.1 Hz, 1H), 8.10 (d, *J* = 7.8 Hz, 1H), 7.97 (s, 1H), 7.96 (s, 1H), 7.56 (dd, *J* = 8.4, 1.6 Hz, 1H), 7.48–7.44 (m, 1H), 7.40 (s, 1H), 7.38 (s, 1H), 7.23–7.18 (m, *J* = 14.9, 5.2 Hz, 3H), 5.52 (s, 2H), 4.34 (q, *J* = 13.8, 6.9 Hz, 2H), 2.37 (s, 3H), 1.41 (t, *J* = 7.2 Hz, 3H) ppm; ^13^C NMR (125 MHz, CDCl_3_) *δ* 166.81 (COO), 143.59, 140.38, 139.94, 129.82, 129.10, 127.79, 126.78, 126.57, 125.92, 123.08, 122.84, 121.26, 120.61, 119.06, 108.63, 108.53, 67.62 (OCH_2_), 37.68 (NCH_2_), 21.71 (CH_3_), 13.87 (CH_3_) ppm; FT-IR: υ_max_ 3052, 2976, 2915, 1711, 1609, 1492, 1472, 1268, 1233, 1177, 1098, 751 cm^−1^; MALDI-TOF (*m*/*z*) calc. for C_23_H_21_NO_2_: 343.426; found [M]^+^: 343.038.

*(9-Ethyl-9H-carbazol-3-yl)methyl benzoate* (**5c**): The compound **5c** was prepared according to *GP-1* from carbazole (50.0 mg, 0.222 mmol, 1.0 mol equiv.), dry DCM (7 mL), dry Et_3_N (0.062 mL, 0.444 mmol, 2.0 mol equiv.), and benzoyl chloride (39 µL, 0.333 mmol, 1.5 mol equiv.) and stirred for 25 h. Purification by silica gel flash column chromatography (hexane:EtOAc 75:25) gave the target product **5c** (55 mg, 76% yield) as a dark-yellow-coloured oil. R*_f_* 0.50 (in hexane:EtOAc 75:25); ^1^H NMR (500 MHz, CDCl_3_) *δ* 8.21 (s, 1H), 8.15–8.07 (m, 3H), 7.59 (dd, *J* = 8.4, 1.5 Hz, 1H), 7.57–7.51 (m, 1H), 7.51–7.46 (m, 1H), 7.45–7.40 (m, 4H), 7.27–7.22 (m, 1H), 5.56 (s, 2H), 4.38 (q, *J* = 7.2 Hz, 2H), 1.44 (t, *J* = 7.2 Hz, 3H) ppm; ^13^C NMR (125 MHz, CDCl_3_) *δ* 166.75 (COO), 140.40, 139.99, 132.95, 130.54, 129.80, 128.39, 126.81, 126.44, 125.96, 123.11, 122.85, 121.33, 120.63, 119.09, 108.65, 108.57, 67.82 (OCH_2_), 37.71 (NCH_2_), 13.88 (CH_3_) ppm; FT-IR: υ_max_ 2970, 1709, 1600, 1494, 1472, 1450, 1370, 1332, 1267, 1234, 1103, 749, 710 cm^−1^; MALDI-TOF (*m*/*z*) calc. for C_22_H_19_NO_2_: 329.399; found [M]^+^: 329.050.

*(9-Ethyl-9H-carbazol-3-yl)methyl 4-bromobenzoate* (**5d**): The compound **5d** was prepared according to *GP-1* from carbazole (50.0 mg, 0.222 mmol, 1.0 mol equiv.), dry DCM (6 mL), dry Et_3_N (0.062 mL, 0.444 mmol, 2.0 mol equiv.), and *p*-bromobenzoyl chloride (73.1 mg, 0.333 mmol, 1.5 mol equiv.) in 1 mL of dry DCM and stirred for 6 h. Purification by silica gel flash column chromatography (hexane:EtOAc 75:25) gave the target product **5d** (21 mg, 24% yield) as a yellow-coloured oil. R*_f_* 0.40 (in EtOAc:hexane 75:25); ^1^H NMR (500 MHz, CDCl_3_) *δ* 8.19 (d, *J* = 1.1 Hz, 1H), 8.11 (d, *J* = 7.8 Hz, 1H), 7.96–7.91 (m, 2H), 7.58–7.52 (m, 3H), 7.51–7.46 (m, 1H), 7.41 (d, *J* = 8.4 Hz, 2H), 7.25 (dd, *J* = 10.7, 4.1 Hz, 1H), 5.53 (s, 2H), 4.36 (q, *J* = 7.2 Hz, 2H), 1.43 (t, *J* = 7.2 Hz, 3H) ppm; ^13^C NMR (125 MHz, CDCl_3_) *δ* 165.98 (COO), 140.39, 140.01, 131.71, 131.31, 129.42, 128.05, 126.84, 126.10, 126.01, 123.11, 122.78, 121.42, 120.60, 119.13, 108.67, 108.59, 68.11 (OCH_2_), 37.69 (NCH_2_), 13.86 (CH_3_) ppm; FT-IR: υ_max_ 2966, 2924, 1716, 1590, 1491, 1472, 1451, 1333, 1264, 1233, 1097, 1011, 749 cm^−1^; MALDI-TOF (*m*/*z*) calc. for C_22_H_18_BrNO_2_: 408.295; found [M]^+^: 408.027.

*(9-Ethyl-9H-carbazol-3-yl)methyl 3-bromobenzoate* (**5e**): The compound **5e** was prepared according to *GP-1* from carbazole (100.0 mg, 0.444 mmol, 1.0 mol equiv.), dry DCM (16 mL), dry Et_3_N (0.124 mL, 0.888 mmol, 2.0 mol equiv.), and *m*-bromo benzoyl chloride (0.087 mL, 0.666 mmol, 1.5 mol equiv.) and stirred for 4 h. Purification by silica gel flash column chromatography (hexane:EtOAc 90:10) gave the target product **5e** (54 mg, 30% yield) as a white-coloured solid. R*_f_* 0.30 (in hexane:EtOAc 90:10); ^1^H NMR (500 MHz, CDCl_3_) *δ* ^1^H NMR (500 MHz, CDCl_3_) *δ* 8.21–8.20 (m, 1H), 8.19 (d, *J* = 1.1 Hz, 1H), 8.12 (d, *J* = 7.8 Hz, 1H), 8.01 (d, *J* = 7.9 Hz, 1H), 7.66 (ddd, *J* = 8.0, 1.9, 1.0 Hz, 1H), 7.57 (dd, *J* = 8.4, 1.6 Hz, 1H), 7.51–7.46 (m, 1H), 7.43 (d, *J* = 2.6 Hz, 1H), 7.42 (d, *J* = 2.3 Hz, 1H), 7.32–7.28 (m, 1H), 7.26–7.23 (m, 1H), 5.54 (s, 2H), 4.39 (q, *J* = 7.2 Hz, 2H), 1.44 (t, *J* = 7.2 Hz, 3H) ppm; ^13^C NMR (125 MHz, CDCl_3_) *δ* 165.41 (COO), 140.40, 140.05, 135.92, 132.75, 132.45, 129.99, 128.40, 126.95, 126.03, 125.98, 123.11, 122.78, 122.50, 121.55, 120.65, 119.15, 108.68, 108.63, 68.30 (OCH_2_), 37.73 (NCH_2_), 13.91 (CH_3_) ppm; FT-IR: υ_max_ 3067, 2975, 2885, 1795, 1716, 1603, 1569, 1492, 1471, 1328, 1279, 1249, 1231, 1116, 1066, 745 cm^−1^; MALDI-TOF (*m*/*z*) calc. for C_22_H_18_BrNO_2_: 408.031; found [M]^+^: 408.031.

*(9-Ethyl-9H-carbazol-3-yl)methyl 2-bromobenzoate* (**5f**): The compound **5f** was prepared according to *GP-1* from carbazole (100.0 mg, 0.444 mmol, 1.0 mol equiv.), dry DCM (16 mL), dry Et_3_N (0.124 mL, 0.888 mmol, 2.0 mol equiv.), and *o*-bromo benzoyl chloride (0.087 mL, 0.666 mmol, 1.5 mol equiv.) and stirred for 4 h. Purification by silica gel flash column chromatography (hexane:EtOAc 90:10) gave the target product **5f** (40 mg, 22% yield) as a light-brown-coloured oil. R*_f_* 0.27 (in EtOAc:hexane 90:10); ^1^H NMR (500 MHz, CDCl_3_) *δ* 8.22 (d, *J* = 1.1 Hz, 1H), 8.11 (d, *J* = 7.7 Hz, 1H), 7.82–7.78 (m, 1H), 7.65 (dd, *J* = 7.5, 1.6 Hz, 1H), 7.59 (dd, *J* = 8.4, 1.6 Hz, 1H), 7.51–7.46 (m, 1H), 7.44–7.41 (m, 2H), 7.35–7.28 (m, 2H), 7.25–7.23 (m, 1H), 5.56 (s, 2H), 4.39 (q, *J* = 7.2 Hz, 2H), 1.44 (t, *J* = 7.2 Hz, 3H) ppm; ^13^C NMR (125 MHz, CDCl_3_) *δ* 166.28 (COO), 140.38, 140.04, 134.39, 132.56, 131.51, 127.58, 127.20, 127.03, 125.99, 125.86, 123.09, 122.83, 121.85, 121.61, 120.64, 119.12, 108.67, 108.59, 68.51 (OCH_2_), 37.73 (NCH_2_), 13.90 (CH_3_) ppm; FT-IR: υ_max_ 3053, 2976, 2930, 2889, 1798, 1725, 1603, 1590, 1492, 1471, 1329, 1286, 1246, 1232, 1128, 1104, 1027, 745 cm^−1^; MALDI-TOF (*m*/*z*) calc. for C_22_H_18_BrNO_2_: 408.295; found [M]^+^: 408.031.

*(9-Ethyl-9H-carbazol-3-yl)methyl 4-nitrobenzoate* (**5g**): The compound **5g** was prepared according to *GP-1* from carbazole (50.0 mg, 0.222 mmol, 1.0 mol equiv.), dry DCM (6 mL), dry Et_3_N (0.062 mL, 0.444 mmol, 2.0 mol equiv.), and *p*-nitrobenzoyl chloride (61.8 mg, 0.333 mmol, 1.5 mol equiv.) in 1 mL of dry DCM and stirred for 13 h. Purification by silica gel flash column chromatography (hexane:EtOAc 75:25) gave the target product **5g** (70 mg, 85% yield) as a bright-yellow-coloured solid. R*_f_* 0.50 (in EtOAc:hexane 75:25); ^1^H NMR (500 MHz, CDCl_3_) *δ* 8.30–8.22 (m, 4H), 8.21 (s, 1H), 8.12 (d, *J* = 7.8 Hz, 1H), 7.58 (d, *J* = 8.3 Hz, 1H), 7.53–7.47 (m, 1H), 7.46–7.40 (m, 2H), 7.29–7.22 (m, 1H), 5.59 (s, 2H), 4.39 (q, *J* = 7.2 Hz, 2H), 1.45 (t, *J* = 7.2 Hz, 3H) ppm; ^13^C NMR (125 MHz, CDCl_3_) *δ* 164.82 (COO), 150.58, 140.43, 140.13, 135.93, 130.90, 126.98, 126.14, 125.57, 123.57, 123.16, 122.72, 121.66, 120.61, 119.24, 108.74, 108.69, 68.85 (OCH_2_), 37.74 (NCH_2_), 13.89 (CH_3_) ppm; FT-IR: υ_max_ 3056, 2952, 1720, 1603, 1525, 1492, 1472, 1346, 1266, 1233, 1098, 718 cm^−1^; MALDI-TOF (*m*/*z*) calc. for C_22_H_18_N_2_O_4_: 374.396; found [M]^+^: 374.002.

*(9-Ethyl-9H-carbazol-3-yl)methyl furan-2-carboxylate* (**5h**): The compound **5h** was prepared according to *GP-1* from carbazole (100.0 mg, 0.444 mmol, 1.0 mol equiv.), dry DCM (16 mL), dry Et_3_N (0.124 mL, 0.888 mmol, 2.0 mol equiv.), and furan-2-carbonyl chloride (66 µL, 0.666 mmol, 1.5 mol equiv.) and stirred for 18 h. Purification by silica gel flash column chromatography (hexane:DCM 33:66) gave the target product **5h** (63 mg, 44% yield) as a colourless oil. R*_f_* 0.10 (in hexane:DCM 33:66); ^1^H NMR (500 MHz, CDCl_3_) *δ* 8.20 (d, *J* = 1.2 Hz, 1H), 8.11 (d, *J* = 7.7 Hz, 1H), 7.59–7.55 (m, 2H), 7.50–7.46 (m, 1H), 7.43–7.39 (m, 2H), 7.25–7.22 (m, 1H), 7.20 (dd, *J* = 3.5, 0.7 Hz, 1H), 6.48 (dd, *J* = 3.5, 1.7 Hz, 1H), 5.53 (s, 2H), 4.38 (q, *J* = 7.2 Hz, 2H), 1.44 (t, *J* = 7.2 Hz, 3H) ppm; ^13^C NMR (125 MHz, CDCl_3_) *δ* 158.89 (COO), 146.35, 144.94, 140.40, 140.06, 127.03, 125.98, 123.12, 122.84, 121.64, 120.65, 119.12, 118.09, 111.88, 108.66, 108.59, 67.72 (OCH_2_), 37.71 (NCH_2_), 13.88 (CH_3_) ppm; FT-IR: υ_max_ 2975, 1714, 1603, 1580, 1492, 1472, 1329, 1289, 1231, 1176, 1167, 1109, 761, 748 cm^−1^; MALDI-TOF (*m/z*) calc. for C_20_H_17_NO_3_: 319.360; found [M]^+^: 319.012.

*(9-Ethyl-9H-carbazol-3-yl)methyl thiophene-2-carboxylate* (**5i**): The compound **5i** was prepared according to *GP-1* from carbazole (100.0 mg, 0.444 mmol, 1.0 mol equiv.), dry DCM (16 mL), dry Et_3_N (0.124 mL, 0.888 mmol, 2.0 mol equiv.), and thiophene-2-carbonyl chloride (71 µL, 0.666 mmol, 1.5 mol equiv.) and stirred for 11 h. Purification by silica gel flash column chromatography (hexane:DCM 50:50) gave the target product **5i** (55 mg, 37% yield) as a colourless oil. R*_f_* 0.30 (in hexane:DCM 50:50); ^1^H NMR (500 MHz, CDCl_3_) *δ* 8.19 (d, *J* = 1.1 Hz, 1H), 8.11 (dd, *J* = 9.7, 6.0 Hz, 1H), 7.83 (dd, *J* = 3.8, 1.2 Hz, 1H), 7.57 (dd, *J* = 8.4, 1.6 Hz, 1H), 7.54 (dd, *J* = 5.0, 1.2 Hz, 1H), 7.51–7.45 (m, 1H), 7.44–7.39 (m, 2H), 7.26–7.22 (m, 1H), 7.08 (dd, *J* = 4.9, 3.8 Hz, 1H), 5.52 (s, 2H), 4.38 (q, *J* = 7.2 Hz, 2H), 1.44 (t, *J* = 7.2 Hz, 3H) ppm; ^13^C NMR (125 MHz, CDCl_3_) *δ* 162.40 (COO), 140.40, 140.01, 134.16, 133.56, 132.42, 127.77, 126.86, 126.22, 125.96, 123.08, 122.85, 121.40, 120.63, 119.10, 108.65, 108.56, 67.90 (OCH_2_), 37.71 (NCH_2_), 13.89 (CH_3_) ppm; FT-IR: υ_max_ 3052, 2971, 1703, 1603, 1524, 1492, 1471, 1417, 1273, 1255, 1232, 1087, 1072, 747, 723 cm^−1^; MALDI-TOF (*m*/*z*) calc. for C_20_H_17_NO_2_S: 335.421; found [M]^+^: 335.039.

*(9-Ethyl-9H-carbazol-3-yl)methyl 1-methyl-1H-indole-2-carboxylate* (**5j**): The compound **5j** was prepared according to *GP-1* from carbazole (50.0 mg, 0.222 mmol, 1.0 mol equiv.), dry DCM (7 mL), dry Et_3_N (0.062 mL, 0.444 mmol, 2.0 mol equiv.), and 1-methyl-1*H*-indole-2-carbonyl chloride (65 mg, 0.333 mmol, 1.5 mol equiv.) and stirred for 24 h. Purification by washing with hexane (2 mL × 5 times) gave the target product **5j** (44 mg, 52% yield) as a white-coloured solid. R*_f_* 0.33 (in hexane:DCM 66:33); ^1^H NMR (500 MHz, CDCl_3_) *δ* 8.22 (d, *J* = 1.0 Hz, 1H), 8.13 (d, *J* = 7.8 Hz, 1H), 7.65 (d, *J* = 8.0 Hz, 1H), 7.60 (dd, *J* = 8.4, 1.6 Hz, 1H), 7.51–7.46 (m, 1H), 7.45–7.41 (m, 2H), 7.40–7.32 (m, 3H), 7.26–7.23 (m, 1H), 7.15–7.11 (m, 1H), 5.55 (s, 2H), 4.39 (q, *J* = 7.2 Hz, 2H), 4.10 (s, 3H), 1.45 (t, *J* = 7.2 Hz, 3H) ppm; ^13^C NMR (125 MHz, CDCl_3_) *δ* 162.33 (COO), 140.38, 139.96, 139.77, 128.02, 126.72, 126.41, 125.96, 125.02, 123.09, 122.83, 122.66, 121.24, 120.64, 120.57, 119.08, 113.51, 110.55, 110.30, 108.65, 108.60, 67.32 (OCH_2_), 37.72 (NCH_2_), 31.75 (NCH_3_), 13.90 (CH_3_) ppm; FT-IR: υ_max_ 3052, 2978, 2946, 2900, 1703, 1467, 1326, 1244, 1232, 1210, 1127, 1077, 816, 747, 731 cm^−1^; MALDI-TOF (*m*/*z*) calc. for C_25_H_22_N_2_O_2_: 382.463; found [M]^+^: 382.006.

*General Procedure-2 (GP-2)—synthesis of bis-ester carbazole compounds* (**7a**–**j**): A reaction flask was charged with carbazole (1.0 mol equiv.) and dry DCM, then cooled to 0 °C under argon atmosphere. To this, dry Et_3_N (3.0 mol equiv.) was added via dropwise portion, then a solution of acyl chloride (3.0 mol equiv.) in dry DCM was added and stirred at 0 °C for 20 min. The reaction mixture was then warmed up to room temperature and stirred for 2–24 h. The resulting reaction mixture was then poured into water and extracted. The organic phase was separated, the inorganic phase was further extracted with DCM, then all the organic phases were combined and washed with aqueous 1M HCl solution followed by *sat.* aqueous NaHCO_3_ solution. The organic phases were then combined, washed with brine, dried over Na_2_SO_4_, filtered, and concentrated under reduced pressure. Purification by silica gel flash column chromatography gave the target product **7a**–**j**.

*(9-Ethyl-9H-carbazole-3,6-diyl)bis(methylene) bis(4-methoxybenzoate)* (**7a**): The compound **7a** was prepared according to *GP-2* from carbazole (50.0 mg, 0.196 mmol, 1.0 mol equiv.), dry DCM (7 mL), dry Et_3_N (0.082 mL, 0.588 mmol, 3.0 mol equiv.), and *p*-methoxy benzoyl chloride (100 mg, 0.588 mmol, 3.0 mol equiv.) in 1 mL of dry DCM and stirred for 2 h. Purification by silica gel flash column chromatography (DCM:EtOH 95:5) gave the target product **7a** (84 mg, 82% yield) as a brown-coloured oil. R*_f_* 0.41 (in hexane:EtOAc 75:25); ^1^H NMR (500 MHz, CDCl_3_) *δ* 8.21 (s, 2H), 8.07–8.03 (m, 4H), 7.58 (d, *J* = 8.4 Hz, 2H), 7.41 (d, *J* = 8.4 Hz, 2H), 6.90 (d, *J* = 8.9 Hz, 4H), 5.52 (s, 4H), 4.36 (q, *J* = 7.1 Hz, 2H), 3.84 (s, 6H), 1.43 (t, *J* = 7.2 Hz, 3H) ppm; ^13^C NMR (125 MHz, CDCl_3_) δ 166.45 (COO), 163.39, 140.26, 131.80, 126.97, 126.91, 122.89, 122.87, 121.30, 113.62, 108.66, 67.39 (OCH_2_), 55.44 (OCH_3_), 37.77 (NCH_2_), 13.86 (CH_3_) ppm; FT-IR: υ_max_ 3048, 2962, 2932, 1701, 1607, 1511, 1270, 1256, 1236, 1168, 1093, 765 cm^−1^; MALDI-TOF (*m*/*z*) calc. for C_32_H_29_NO_6_: 523.585; found [M]^+^: 523.238.

*(9-Ethyl-9H-carbazole-3,6-diyl)bis(methylene) bis(4-methylbenzoate)* (**7b**): The compound **7b** was prepared according to *GP-2* from carbazole (50.0 mg, 0.196 mmol, 1.0 mol equiv.), dry DCM (7 mL), dry Et_3_N (0.082 mL, 0.588 mmol, 3.0 mol equiv.), and *p*-methyl benzoyl chloride (91 mg, 0.588 mmol, 3.0 mol equiv.) in 1 mL of dry DCM and stirred for 2 h. Purification by silica gel flash column chromatography (hexane:EtOAc 85:15) gave the target product **7b** (77 mg, 80% yield) as a yellow-coloured solid. R*_f_* 0.59 (in hexane:EtOAc 75:25); ^1^H NMR (500 MHz, CDCl_3_) *δ* 8.21 (s, 2H), 7.99 (d, *J* = 8.2 Hz, 4H), 7.59 (dd, *J* = 8.4, 1.4 Hz, 2H), 7.41 (d, *J* = 8.4 Hz, 2H), 7.22 (d, *J* = 8.0 Hz, 4H), 5.53 (s, 4H), 4.37 (q, *J* = 7.1 Hz, 2H), 2.39 (s, 6H), 1.43 (t, *J* = 7.2 Hz, 3H) ppm; ^13^C NMR (125 MHz, CDCl_3_) δ 166.80 (COO), 143.62, 140.30, 129.82, 129.11, 127.72, 127.00, 126.83, 122.91, 121.35, 108.69, 67.53 (OCH_2_), 37.80 (NCH_2_), 21.72 (CH_3_), 13.89 (CH_3_) ppm; FT-IR: υ_max_ 3048, 2958, 2924, 1715, 1610, 1497, 1365, 1267, 1237, 1100, 749 cm^−1^; MALDI-TOF (*m/z*) calc. for C_32_H_29_NO_4_: 491.587; found [M]^+^: 491.011.

*(9-Ethyl-9H-carbazole-3,6-diyl)bis(methylene) dibenzoate* (**7c**): The compound **7c** was prepared according to *GP-2* from carbazole (50.0 mg, 0.196 mmol, 1.0 mol equiv.), dry DCM (7 mL), dry Et_3_N (0.082 mL, 0.588 mmol, 3.0 mol equiv.), and benzoyl chloride (83 mg, 0.588 mmol, 3.0 mol equiv.) in 1 mL of dry DCM and stirred for 2 h. Purification by silica gel flash column chromatography (hexane:EtOAc 75:25) gave the target product **7c** (47 mg, 51% yield) as a yellow-coloured solid. R*_f_* 0.56 (in hexane:EtOAc 75:25); ^1^H NMR (500 MHz, CDCl_3_) *δ* 8.22 (s, 2H), 8.09 (d, *J* = 7.3 Hz, 4H), 7.59 (dd, *J* = 8.5, 1.0 Hz, 2H), 7.57–7.51 (m, 2H), 7.45–7.39 (m, 6H), 5.55 (s, 4H), 4.38 (q, *J* = 7.2 Hz, 2H), 1.44 (t, *J* = 7.2 Hz, 3H) ppm; ^13^C NMR (125 MHz, CDCl_3_) δ 166.75 (COO), 140.35, 132.99, 130.28, 129.81, 128.57, 128.40, 127.08, 126.73, 122.93, 121.44, 108.73, 67.74 (OCH_2_), 37.84 (NCH_2_), 13.90 (CH_3_) ppm; FT-IR: υ_max_ 3060, 2972, 2950, 2932, 1712, 1604, 1494, 1483, 1266, 1235, 1101, 1067, 801, 710 cm^−1^; MALDI-TOF (*m*/*z*) calc. for C_30_H_25_NO_4_: 463.533; found [M]^+^: 463.020.

*(9-Ethyl-9H-carbazole-3,6-diyl)bis(methylene) bis(4-bromobenzoate)* (**7d**): The compound **7d** was prepared according to *GP-2* from carbazole (50.0 mg, 0.196 mmol, 1.0 mol equiv.), dry DCM (7 mL), dry Et_3_N (0.082 mL, 0.588 mmol, 3.0 mol equiv.), and *p*-bromo benzoyl chloride (129 mg, 0.588 mmol, 3.0 mol equiv.) and stirred for 2 h. Purification by silica gel flash column chromatography (hexane:EtOAc 75:25) gave the target product **7d** (85 mg, 70% yield) as a yellow-coloured solid. R*_f_* 0.66 (in hexane:EtOAc 75:25); ^1^H NMR (500 MHz, CDCl_3_) *δ* 8.20 (s, 2H), 7.94 (d, *J* = 8.5 Hz, 4H), 7.61–7.54 (m, 6H), 7.43 (d, *J* = 8.4 Hz, 2H), 5.53 (s, 4H), 4.38 (q, *J* = 7.1 H, 2Hz), 1.44 (t, *J* = 7.2 Hz, 3H) ppm; ^13^C NMR (125 MHz, CDCl_3_) δ 166.00 (COO), 140.42, 131.99, 131.76, 131.34, 129.37, 128.13, 127.18, 126.48, 122.92, 121.55, 108.81, 68.01 (OCH_2_), 37.87 (NCH_2_), 13.91 (CH_3_) ppm; FT-IR: υ_max_ 3039, 2940, 2883, 1717, 1589, 1396, 1284, 1267, 1169, 1113, 1067, 1011, 752 cm^−1^; MALDI-TOF (*m*/*z*) calc. for C_30_H_23_Br_2_NO_4_: 621.325; found [M-2H]^+^: 619.449.

*(9-Ethyl-9H-carbazole-3,6-diyl)bis(methylene) bis(3-bromobenzoate)* (**7e**): The compound **7e** was prepared according to *GP-2* from carbazole (41.0 mg, 0.161 mmol, 1.0 mol equiv.), dry DCM (6 mL), dry Et_3_N (0.070 mL, 0.482 mmol, 3.0 equiv.), and *m*-bromo benzoyl chloride (0.064 mL, 0.482 mmol, 3.0 mol equiv.) and stirred for 3.5 h. Purification by silica gel flash column chromatography (hexane:DCM 50:50) gave the target product **7e** (68 mg, 67% yield) as a white-coloured solid. R*_f_* 0.43 (in hexane:DCM 50:50); ^1^H NMR (500 MHz, CDCl_3_) *δ* 8.22–8.19 (m, 4H), 8.03–7.99 (m, 2H), 7.68–7.64 (m, 2H), 7.59 (dd, *J* = 8.4, 1.5 Hz, 2H), 7.43 (d, *J* = 8.4 Hz, 2H), 7.32–7.27 (m, 2H), 5.55 (s, 4H), 4.39 (q, *J* = 7.2 Hz, 2H), 1.44 (t, *J* = 7.2 Hz, 3H) ppm; ^13^C NMR (125 MHz, CDCl_3_) *δ* 165.37 (COO), 140.43, 135.93, 132.73, 132.39, 129.98, 128.39, 127.25, 126.35, 122.90, 122.49, 121.64, 108.81, 68.17 (2 × OCH_2_), 37.86 (NCH_2_), 13.91 (CH_3_) ppm; FT-IR: υ_max_ 2967, 1727, 1714, 1568, 1495, 1373, 1323, 1289, 1249, 1238, 1075, 796, 746 cm^−1^; MALDI-TOF (*m*/*z*) calc. for C_30_H_23_Br_2_NO_4_: 621.325; found [M]^+^: 621.027.

*(9-Ethyl-9H-carbazole-3,6-diyl)bis(methylene) bis(2-bromobenzoate)* (**7f**): The compound **7f** was prepared according to *GP-2* from carbazole (200.0 mg, 0.784 mmol, 1.0 mol equiv.), dry DCM (28 mL), dry Et_3_N (0.330 mL, 2.35 mmol, 3.0 mol equiv.), and *o*-bromo benzoyl chloride (307 mL, 2.35 mmol, 3.0 mol equiv.) in 8 mL of dry DCM and stirred for 2 h. Purification by silica gel flash column chromatography (hexane:EtOAc 75:25) gave the target product **7f** (458 mg, 94% yield) as a yellow-coloured solid. R*_f_* 0.46 (in hexane:EtOAc 75:25); ^1^H NMR (500 MHz, CDCl_3_) *δ* 8.22 (s, 2H), 7.81 (d, *J* = 7.5 Hz, 2H), 7.65 (d, *J* = 7.5 Hz, 2H), 7.60 (d, *J* = 8.4 Hz, 2H), 7.42 (d, *J* = 8.4 Hz, 2H), 7.31 (m, 4H), 5.56 (s, 4H), 4.38 (q, *J* = 7.2 Hz, 2H), 1.43 (t, *J* = 7.2 Hz, 3H) ppm; ^13^C NMR (125 MHz, CDCl_3_) *δ* 166.24 (COO), 140.40, 134.38, 132.56, 132.38, 131.50, 127.26, 127.20, 126.21, 122.91, 121.84, 121.64, 108.77, 68.37 (OCH_2_), 37.84 (NCH_2_), 13.89 (CH_3_) ppm; FT-IR: υ_max_ 2985, 2959, 1727, 1634, 1590, 1487, 1430, 1370, 1323, 1241, 1130, 1098, 1043, 1026, 929, 800, 739 cm^−1^; MALDI-TOF (*m*/*z*) calc. for C_30_H_23_Br_2_NO_4_: 621.325; found [M-2H]^+^: 619.195.

*(9-Ethyl-9H-carbazole-3,6-diyl)bis(methylene) bis(4-nitrobenzoate)* (**7g**): The compound **7g** was prepared according to *GP-2* from carbazole (50.0 mg, 0.196 mmol, 1.0 mol equiv.), dry DCM (7 mL), dry Et_3_N (0.082 mL, 0.588 mmol, 3.0 mol equiv.), and *p*-nitro benzoyl chloride (109 mg, 0.588 mmol, 3.0 mol equiv.) and stirred for 5 h. Purification by silica gel flash column chromatography (hexane:EtOAc 50:50) gave the target product **7g** (86 mg, 94% yield) as a yellow-coloured solid. R*_f_* 0.49 (in hexane:EtOAc 75:25); ^1^H NMR (500 MHz, CDCl_3_) *δ* 8.29–8.20 (m, 10H), 7.60 (dd, *J* = 8.3, 1.3 Hz, 2H), 7.45 (d, *J* = 8.4 Hz, 2H), 5.59 (s, 4H), 4.40 (q, *J* = 7.1 Hz, 2H), 1.45 (t, *J* = 7.2 Hz, 3H) ppm; ^13^C NMR (125 MHz, CDCl_3_) δ 164.80 (COO), 150.61, 140.54, 135.84, 130.90, 127.40, 126.06, 123.59, 122.90, 121.77, 108.97, 68.69 (OCH_2_), 37.91 (NCH_2_), 13.92 (CH_3_) ppm; FT-IR: υ_max_ 3112, 3084, 2964, 2928, 2855, 1717, 1609, 1522, 1268, 1240, 1096 cm^−1^; MALDI-TOF (*m*/*z*) calc. for C_30_H_23_N_3_O_8_: 553.527; found [M]^+^: 553.063.

*(9-Ethyl-9H-carbazole-3,6-diyl)bis(methylene) bis(furan-2-carboxylate)* (**7h**): The compound **7h** was prepared according to *GP-2* from carbazole (50.0 mg, 0.196 mmol, 1.0 mol equiv.), dry DCM (7 mL), dry Et_3_N (0.082 mL, 0.588 mmol, 3.0 mol equiv.), and then furan-2-carbonyl chloride (58 µL, 0.588 mmol, 3.0 mol equiv.) and stirred for 3 h. Purification by silica gel flash column chromatography (hexane:DCM 33:66) gave the target product **7h** (65 mg, 74% yield) as a white-coloured solid. R*_f_* 0.19 (in hexane:DCM 33:66); ^1^H NMR (500 MHz, CDCl_3_) *δ* 8.20 (s, 2H), 7.60–7.54 (m, 4H), 7.40 (d, *J* = 8.4 Hz, 2H), 7.20 (d, *J* = 3.3 Hz, 2H), 6.48 (dd, *J* = 3.5, 1.7 Hz, 2H), 5.52 (s, 4H), 4.36 (q, *J* = 7.2 Hz, 2H), 1.42 (t, *J* = 7.2 Hz, 3H) ppm; ^13^C NMR (125 MHz, CDCl_3_) *δ* 158.83 (COO), 146.35, 144.88, 140.41, 127.23, 126.31, 122.93, 121.65, 118.11, 111.87, 108.73, 67.56 (OCH_2_), 37.80 (NCH_2_), 13.85 (CH_3_) ppm; FT-IR: υ_max_ 2970, 1711, 1580, 1474, 1287, 1237, 1167, 1106, 1077, 950, 885, 795, 766, 755 cm^−1^; MALDI-TOF (*m*/*z*) calc. for C_26_H_21_NO_6_: 443.455; found [M]^+^: 443.014.

*(9-Ethyl-9H-carbazole-3,6-diyl)bis(methylene) bis(thiophene-2-carboxylate)* (**7i**): The compound **7i** was prepared according to *GP-2* from carbazole (50.0 mg, 0.196 mmol, 1.0 mol equiv.), dry DCM (7 mL), dry Et_3_N (0.082 mL, 0.588 mmol, 3.0 mol equiv.), and thiophene-2-carbonyl chloride (63 µL, 0.588 mmol, 3.0 mol equiv.) and stirred for 5 h. Purification by silica gel flash column chromatography (hexane:DCM 66:33) gave the target product **7i** (73 mg, 78% yield) as a white-coloured solid. R*_f_* 0.14 (in hexane:DCM 66:33); ^1^H NMR (500 MHz, CDCl_3_) *δ* 8.20 (s, 2H), 7.83 (dd, *J* = 3.8, 1.2 Hz, 2H), 7.58 (dd, *J* = 8.4, 1.6 Hz, 2H), 7.54 (dd, *J* = 5.0, 1.2 Hz, 2H), 7.41 (d, *J* = 8.4 Hz, 2H), 7.08 (dd, *J* = 4.9, 3.8 Hz, 2H), 5.52 (s, 4H), 4.38 (q, *J* = 7.2 Hz, 2H), 1.43 (t, *J* = 7.2 Hz, 3H) ppm; ^13^C NMR (125 MHz, CDCl_3_) *δ* 162.38 (COO), 140.39, 134.13, 133.60, 132.45, 127.78, 127.11, 126.55, 122.94, 121.49, 108.74, 67.80 (OCH_2_), 37.85 (NCH_2_), 13.90 (CH_3_) ppm; FT-IR: υ_max_ 2972, 1705, 1498, 1486, 1417, 1371, 1272, 1256, 1236, 1084, 1073, 1032, 891, 798, 747, 723 cm^−1^; MALDI-TOF (*m*/*z*) calc. for C_26_H_21_NO_4_S_2_: 475.577; found [M]^+^: 475.028.

*(9-Ethyl-9H-carbazole-3,6-diyl)bis(methylene) bis(1-methyl-1H-indole-2-carboxylate)* (**7j**): The compound **7j** was prepared according to *GP-2* from carbazole (50.0 mg, 0.196 mmol, 1.0 mol equiv.), dry DCM (7 mL), dry Et_3_N (0.082 mL, 0.588 mmol, 3.0 mol equiv.), and 1-methyl-1*H*-indole-2-carbonyl chloride (114 mg, 0.588 mmol, 3.0 mol equiv.) in 1 mL of dry DCM and stirred for 24 h. Purification by silica gel flash column chromatography (hexane:DCM 25:75) gave the target product **7j** (46 mg, 41% yield) as an orange-coloured solid. R*_f_* 0.42 (in hexane:DCM 25:75); ^1^H NMR (500 MHz, CDCl_3_) *δ* 8.23 (s, 2H), 7.64 (d, *J* = 8.0 Hz, 2H), 7.60 (dd, *J* = 8.4, 1.5 Hz, 2H), 7.43 (d, *J* = 8.4 Hz, 2H), 7.40–7.31 (m, 6H), 7.15–7.10 (m, 2H), 5.55 (s, 4H), 4.39 (q, *J* = 7.2 Hz, 2H), 4.10 (s, 6H), 1.45 (t, *J* = 7.2 Hz, 3H).ppm; ^13^C NMR (125 MHz, CDCl_3_) *δ* 162.31 (COO), 140.37, 139.81, 128.01, 126.97, 126.78, 125.99, 125.05, 122.97, 122.67, 121.34, 120.58, 110.58, 110.30, 108.77, 67.22 (OCH_2_), 37.86 (NCH_2_), 31.75 (NCH_3_), 13.91 (CH_3_) ppm; FT-IR: υ_max_ 2953, 1704, 1611, 1517, 1467, 1399, 1321, 1247, 1217, 1146, 1077, 746 cm^−1^; MALDI-TOF (*m*/*z*) calc. for C_36_H_31_N_3_O_4_: 569.661; found [M]^+^: 569.150.

### 3.2. Biological Studies

#### 3.2.1. α-Glucosidase Inhibition Assay

The α-glucosidase inhibitory activity was performed using the method described by Schmidt et al. [25]. Since enzyme inhibition results may vary depending on experimental parameters, such as enzyme source, substrate concentration, buffer composition, and detection method, Acarbose was evaluated under the same assay conditions as the tested compounds to allow reliable relative comparison within the experimental system. In brief, 90 mm^3^ of 0.1 M phosphate buffer (pH 7.5, 0.02% NaN_3_), a 10 mm^3^ test sample dissolved in DMSO, and 80 mm^3^ of enzyme solution (well concentration of 0.05 U/cm^3^) were added to each well. The lead compounds, including 10b, were fully soluble in the assay buffers and remained stable throughout the *in vitro* enzyme inhibition experiments, ensuring reliable activity measurements. The mixture was incubated at 28 °C for 10 min before adding PNPG to a final volume of 200 mm^3^ (final well concentration of 1.0 mM). A blank was used consisting of the enzyme, substrate, and test solvent instead of the sample. Absorbance was measured at 405 nm every 40 s for 35 min. A BioTek Power Wave XS microplate photometer (Biotek*^®^* Instruments, Winooski, VT, USA) with a built-in incubator and controlled by the programme GEN5 9ver. 2.05.20050 (BioTek Instruments, Winooski, VT, USA) was used for the incubation and absorbance measurements. The α-glucosidase inhibitory activity was expressed as percentage inhibition and calculated using the following formula:

Inhibition % = (Slopeblank − Slopesample)/Slopeblank × 100(1)

Acarbose was used as the positive control, and all measurements were performed in triplicate (Student’s *t*-test with *p* < 0.05 showing significance).

#### 3.2.2. α-Amylase Inhibition Assay

The α-amylase inhibitory activity was measured using the method described by Okutan et al. with the minor changes [26]. In brief, 80 mm^3^ of 0.1 M phosphate buffer (pH 6.0, 0.02% NaN_3_), a 20 mm^3^ test sample dissolved in DMSO, and 80 mm^3^ of enzyme solution (well concentration of 0.05 U/cm^3^) were added to each well. After incubation at 37 °C for 10 min, the reaction was started by adding CNP-G3 to a final volume of 200 mm^3^ (final well concentration of 1.0 mM). A blank was used consisting of the enzyme, substrate, and test solvent instead of the sample. Absorbance was measured at 405 nm at for 30m. A background sample consisting of enzyme, sample, and buffer in place of the substrate for each compound was employed to eliminate the effect of the absorbance for analytes that absorbs at 405 nm. The BioTek Power Wave XS microplate photometer with built-in incubator controlled by the programme GEN5 (ver. 2.05.2005) was used for the incubation and absorbance measurements. The α-amylase inhibitory activity was expressed as percentage inhibition ± SD and calculated using the following formula:

Inhibition % = (Ablank − Asample)/Ablank × 100(2)

Acarbose was used as the positive control, and all measurements were performed in triplicate (Student’s *t*-test with *p* < 0.05 showing significance).

All IC_50_ values were calculated by GraphPad Prism (8.0.1) using non-linear regression ± standard deviation.

### 3.3. Molecular Modelling

The crystallographic structures of α-amylase and α-glucosidase were obtained from the Protein Data Bank [27], with the PDB codes 1B2Y [28] (α-amylase, resolution 3.20 Å) and 3W37 [29] (α-glucosidase, resolution 1.70 Å). The crystal structures were prepared using BIOVIA Discovery Studio Visualizer 2025 (Dassault Systèmes, San Diego, CA, USA)**,** where crystallographic water molecules and other heteroatoms were removed, retaining only the amino acid residues. Subsequently, MGLTools was employed to add missing hydrogen atoms, assign atomic charges, and prepare the receptor structures for docking calculations.

#### 3.3.1. Ligand Preparation

The docked protein–ligand complexes were visualized and analyzed using BIOVIA Discovery Studio Visualizer (DSV). Two-dimensional and three-dimensional interaction diagrams were generated using DSV to identify and illustrate key intermolecular interactions between the ligands and the active site residues of the target proteins [30], and then visualized with the UCSF Chimera software package (v. 1.13) [31]. The geometries of all ligands were optimized using the semiempirical AM1 method through GaussView 5.0.9 [32]. The AM1-BCC atomic partial charges for the ligands were calculated using the Antechamber module implemented in the AMBER v.11 package programme [33].

#### 3.3.2. Docking Study

Molecular docking studies were carried out using DOCK 6.5. Grid boxes were generated by centring them on the active binding sites of the receptors. The grid centre coordinates for α-amylase were set to x = 21.279, y = 8.458, and z = 48.457, while for α-glucosidase they were x = −1.728, y = −4.415, and z = −22.643. The grid box dimensions were defined as 43.437 × 41.279 × 42.442 Å for α-amylase and 40.000 × 40.000 × 40.000 Å for α-glucosidase.

The UCSF Chimera package [31] was employed for the preparation of the protein and the co-crystalized ligand to validate docking processes. The spheres in the active site, with a 10 Å radius from the crystal structure of the co-crystalized ligand, were chosen for gridding and surrounded by a box with coordinates of 10.265, 13.445, 67.457 and dimensions of 22.131 × 24.384 × 26.733 Å. The same grid files were used for the virtual screening. In all docking processes, the flex mode was applied, namely the protein was kept fixed while ligands were allowed to rotate freely. The receptor was treated as rigid during docking, while full conformational flexibility was allowed for ligand rotatable bonds.

The docking protocol was validated by re-docking the co-crystallized ligands into the active sites of the respective enzymes. The resulting RMSD values were 1.164 Å for α-amylase (1B2Y) and 2.217 Å for α-glucosidase (3W37), confirming the reliability of the docking procedure. The scoring function implemented in DOCK 6 includes van der Waals and electrostatic interaction terms, and the calculated binding energies are reported in kcal/mol.

The PDB files corresponding to the docked complexes of the target protein with compound **7g**, Acarbose, and compound **5g** have been provided as Supplementary Files S1–S5.

#### 3.3.3. Absorption, Distribution, Metabolism, Excretion, and Toxicity (ADMET) Prediction

The ADME properties of ligands by SwissADME web service were also defined to identify the physicochemical and drug-likeness properties of the ligands. The levels of gastrointestinal absorption, blood–brain barrier (BBB) penetration, bioavailability, and inhibition efficiency for a range of hemeproteins, namely CYP1A2, CYP2C19, CYP2C9, CYP2D6, and CYP3A4 enzymes, were identified as physicochemical properties of the compounds.

## 4. Conclusions

In conclusion, new series of carbazole-based mono- and bis-ester compounds were successfully prepared and evaluated for their anti-diabetic potential. The biological assessment revealed predominantly weak to moderate inhibition against α-amylase, while several derivatives exhibited more pronounced and selective activity toward α-glucosidase, with **7g** standing out as the most active compound, even exceeding Acarbose. Docking studies were in good agreement with the experimental results, supporting the binding preference of active compounds within the enzyme active sites, especially for **7g**. In addition, ADME analysis suggested generally acceptable pharmacokinetic properties, although limited gastrointestinal absorption was predicted. Overall, these findings indicate that carbazole-based ester frameworks represent a promising platform for further development of α-glucosidase inhibitors.

## Figures and Tables

**Table 1:** α-Glucosidase and α-amylase enzyme inhibition values.

	α-Glucosidase	α-Amylase
Compounds	IC_50_ μM *	
**5a**	>800	>800
**5b**	>800	>800
**5c**	>800	>800
**5d**	>800	>800
**5e**	>800	>800
**5f**	>800	>800
**5g**	>800	635.3 ± 1.51
**5h**	>800	>800
**5i**	>800	>800
**5j**	>800	>800
**7a**	54.06 ± 7.70	593.7 ± 13.40
**7b**	>800	>800
**7c**	>800	>800
**7d**	>800	511.5 ± 4.88
**7e**	>800	>800
**7f**	614 ± 1.91	>800
**7g**	22.79 ± 7.85	48.08 ± 6.49
**7h**	>800	>800
**7i**	>800	>800
**7j**	475.1 ± 1.96	157.5 ± 3.20
Acarbose **	1033.8 ± 3.4	0.1 ± 2.1 × 10^−7^

* Values are expressed as mean ± SD (n = 3). Different superscript letters indicate statistically significant differences (*p* < 0.05, one-way ANOVA followed by Tukey’s test).** Positive control.

**Table 2:** Scores (DOCK 6 scoring function outputs) of the best ligands **7a**, **7j**, **7g**, **7f,** and **5g**, as well as Acarbose, in binding sites of designated enzymes obtained from molecular docking studies.

Ligands	Dock Score (kcal/mol) α-Amylase	Dock Score (kcal/mol) α-Glucosidase
**7a**	−42.51	−21.01
**7j**	−57.08	−37.67
**7g**	−46.27	−25.60
**7f**	NA	−11.60
**5g**	−36.37	NA
**Acarbose**	−70.03	−62.27

NA: not applicable.

**Table 3:** Predicted ADME properties of the ligand **7g**.

Ligand	GI Absorption	BBB Permeant	Bioavailability	CYP1A2	CYP2C19	CYP2C9	CYP2D6	CYP3A4
**7g**	Low	No	0.17	No	No	Yes	No	No

## Data Availability

The data presented in this study are available in the article or Supplementary Materials. Additional crystallographic data with CCDC reference number 2552292 (**5j**) has been deposited within the Cambridge Crystallographic Data Centre via www.ccdc.cam.ac.uk/deposit. URL (accessed on 7 May 2026).

## Supplementary Materials

The following supporting information can be downloaded at: https://www.mdpi.com/article/10.3390/molecules31122113/s1. Figures S1–S40: ^1^H NMR and ^13^C NMR spectra; Table S1: X-ray crystallographic data and structure refinement. Supplementary Files S1–S5: Material and Methods for Docking study.
