# Supplementary material for: Carbazole-Based Ester Derivatives as Potential α-Glucosidase Inhibitors; Synthesis, Biological Evaluation, and Molecular Docking Studies
Source: Molecules. 2026 Jun 16;31(12):2113. doi: 10.3390/molecules31122113 (PMC13306028; doi:10.3390/molecules31122113)
Supplement: Supplementary file 1 [file molecules-31-02113-s001.zip › molecules-4336174-supplementary.pdf]

# Supporting Information

# Carbazole-Based Ester Derivatives as Potential α-Glucosidase Inhibitors; Synthesis, Biological Evaluation and Molecular Docking Studies

Leyla Kaya[1*], Mehmet F. Saglam[2*], Rabia Sarıbas[2], Murat Bingul[3*], Alev Arslantürk Bingül[4], Mahmut Yıldız[2], Hasan Sahin[5], Sadık Metin Ceyhan[2,6], Ustun Utkan Acar[2], Hakan Kandemir[7], Ibrahim F. Sengul[2]

[1] Department of Pharmaceutical Toxicology, Faculty of Pharmacy, Zonguldak Bulent Ecevit University, Zonguldak, Türkiye

[2] Department of Chemistry, Faculty of Science, Gebze Technical University, Kocaeli, Türkiye

[3] Department of Basic Pharmaceutical Sciences, Faculty of Pharmacy, Dicle University, Diyarbakır, Türkiye

[4] Department of Chemistry, Institute of Natural and Applied Sciences, Dicle University, Diyarbakır, Türkiye

[5] Department of Pharmacognosy, Faculty of Pharmacy, Dicle University, Diyarbakır, Türkiye

[6] Central Laboratory, Kocaeli University, Kocaeli, Türkiye

[7] Department of Chemistry, Faculty of Art and Science, Tekirdag Namık Kemal University, Tekirdag, Türkiye

*Correspondences: leyla.kaya@beun.edu.tr, mfsaglam@gtu.edu.tr, murat.bingul@dicle.edu.tr

**Content:**

1. **$^{1}$H NMR and $^{13}$C NMR Spectra** ………………………………………... S2-S11
2. **X-ray crystallographic data and structure refinement** …………………... S22
3. **Material and Methods for Docking study** ………………………………….. S23

# 1. $^{1}$H NMR and $^{13}$C NMR Spectra

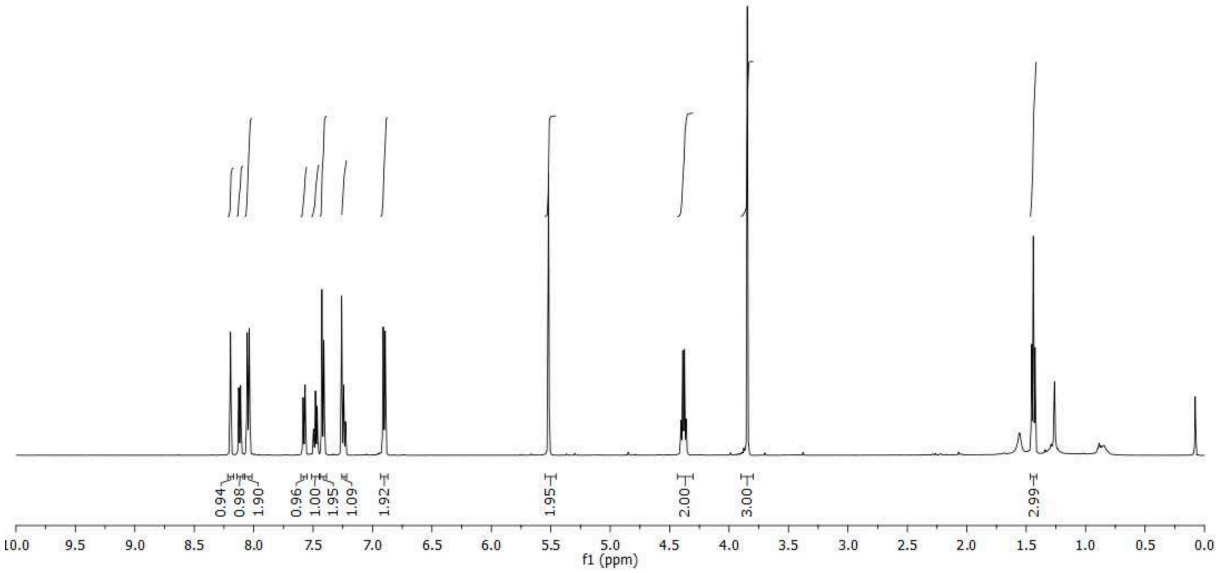


Figure S1: $^{1}$H NMR spectrum of the compound **5a** (500 MHz, in $CDCl_3$)

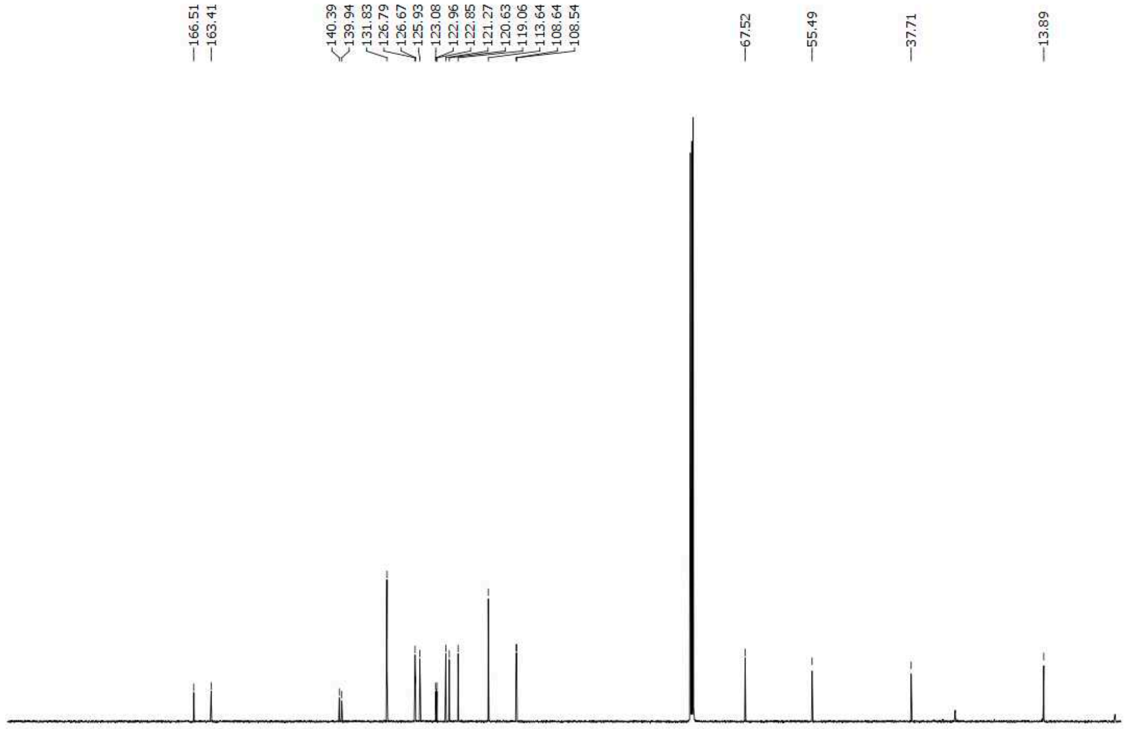


Figure S2: $^{13}$C NMR spectrum of the compound **5a** (125 MHz, in $CDCl_3$)

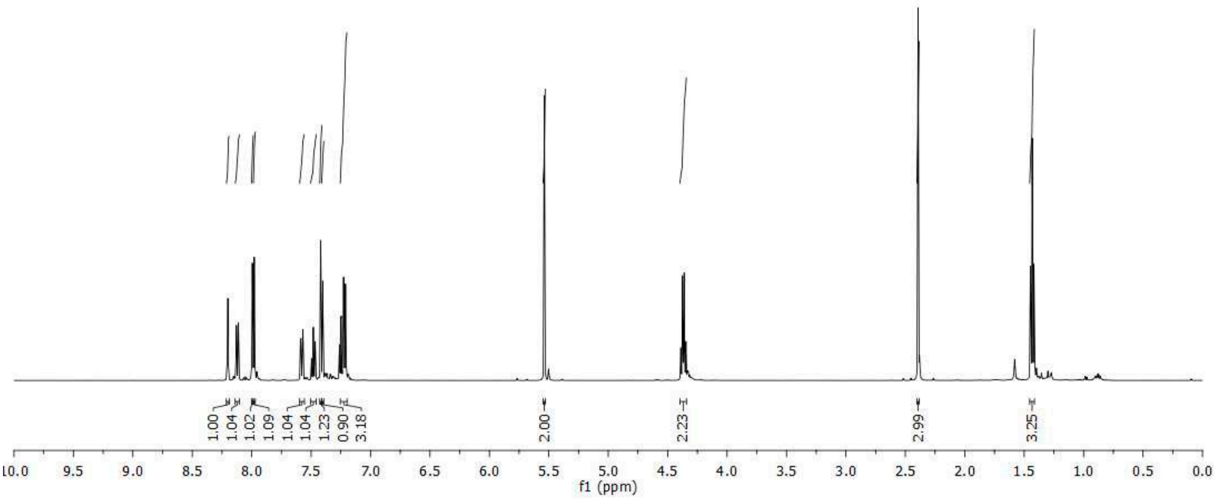


Figure S3: $^{1}$H NMR spectrum of the compound **5b** (500 MHz, in $CDCl_3$)

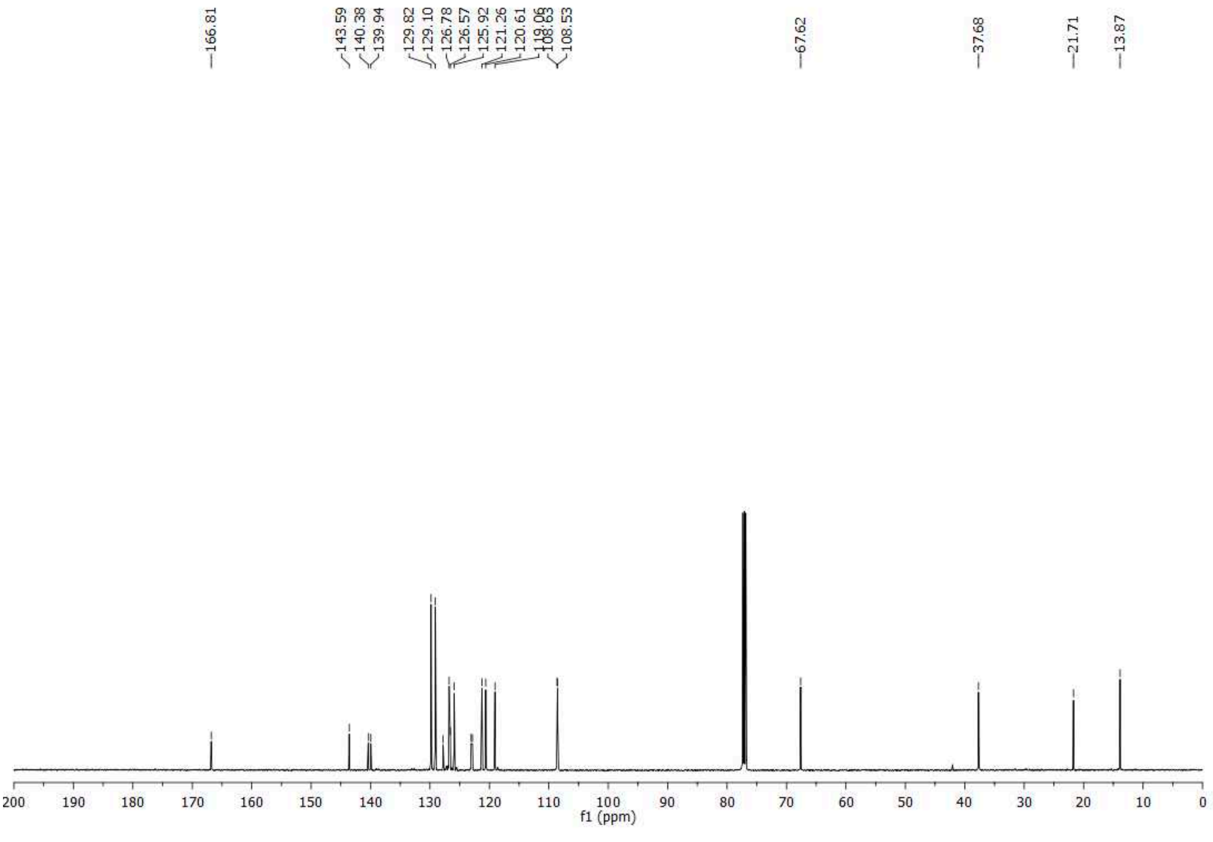


Figure S4: $^{13}$C NMR spectrum of the compound **5b** (125 MHz, in $CDCl_3$)

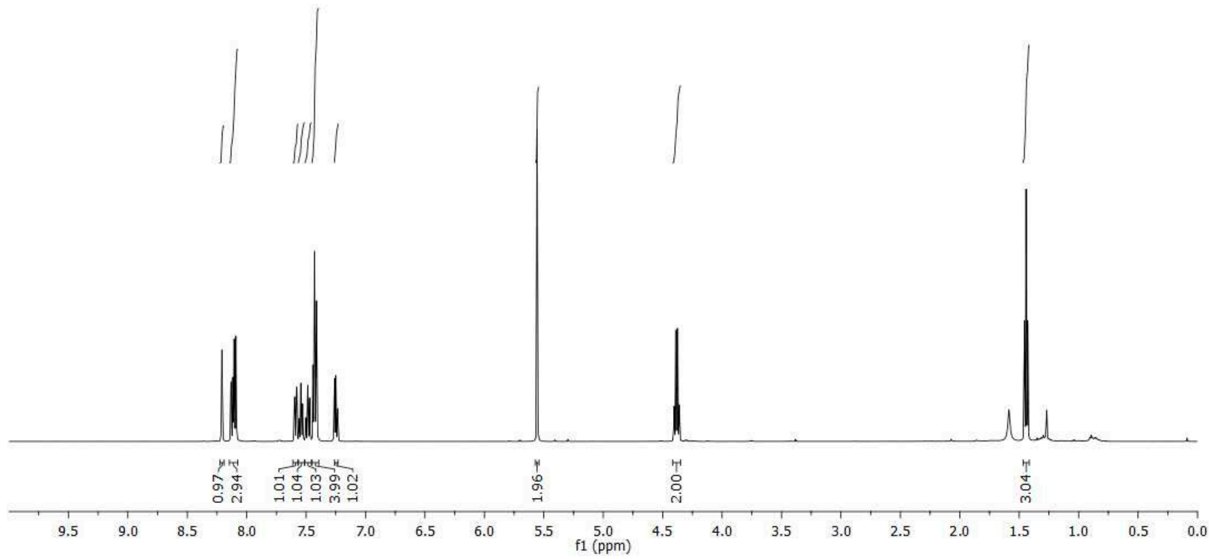


Figure S5: $^{1}$H NMR spectrum of the compound **5c** (500 MHz, in $CDCl_3$)

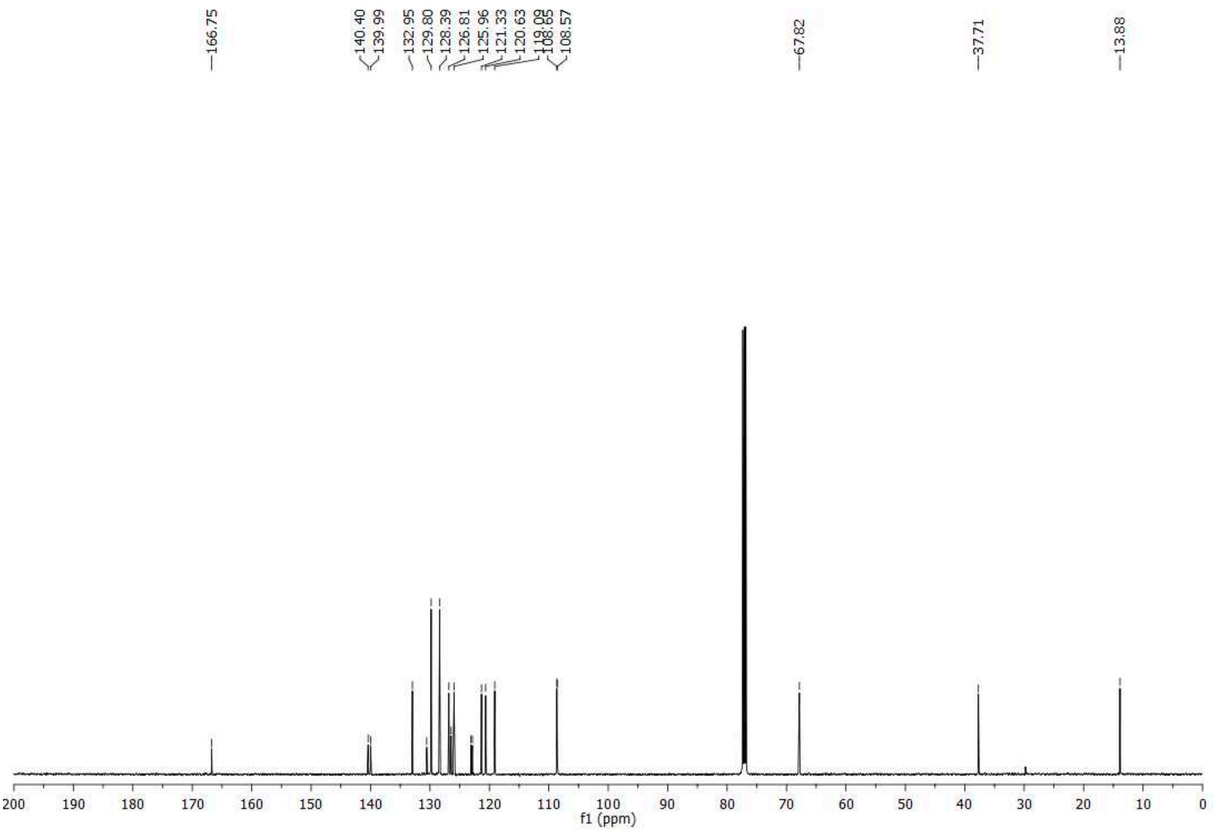


Figure S6: $^{13}$C NMR spectrum of the compound **5c** (125 MHz, in $CDCl_3$)

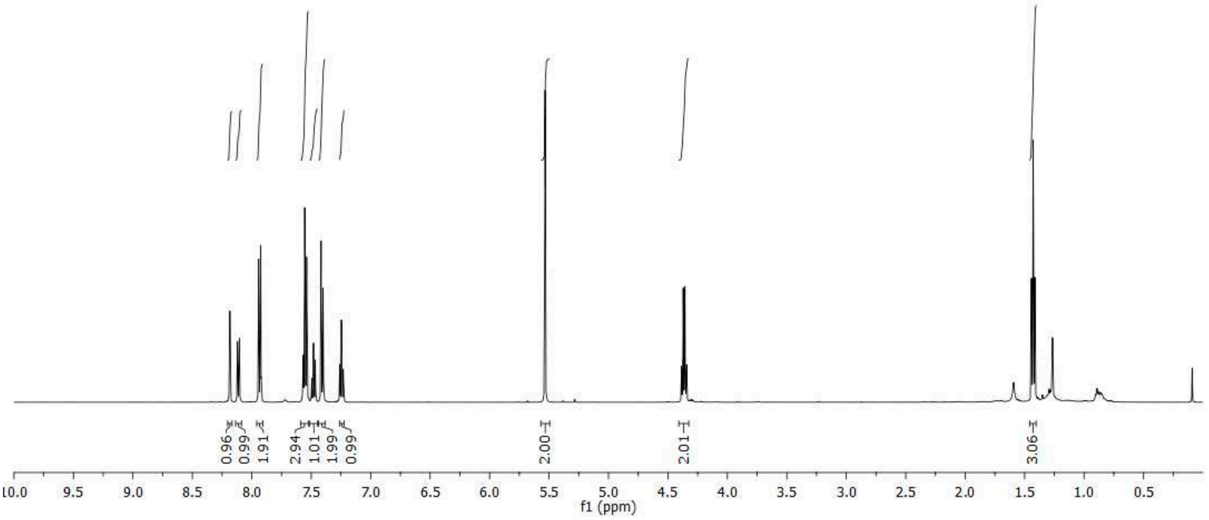

Figure S7: $^{1}H$ NMR spectrum of the compound **5d** (500 MHz, in $CDCl_3$)

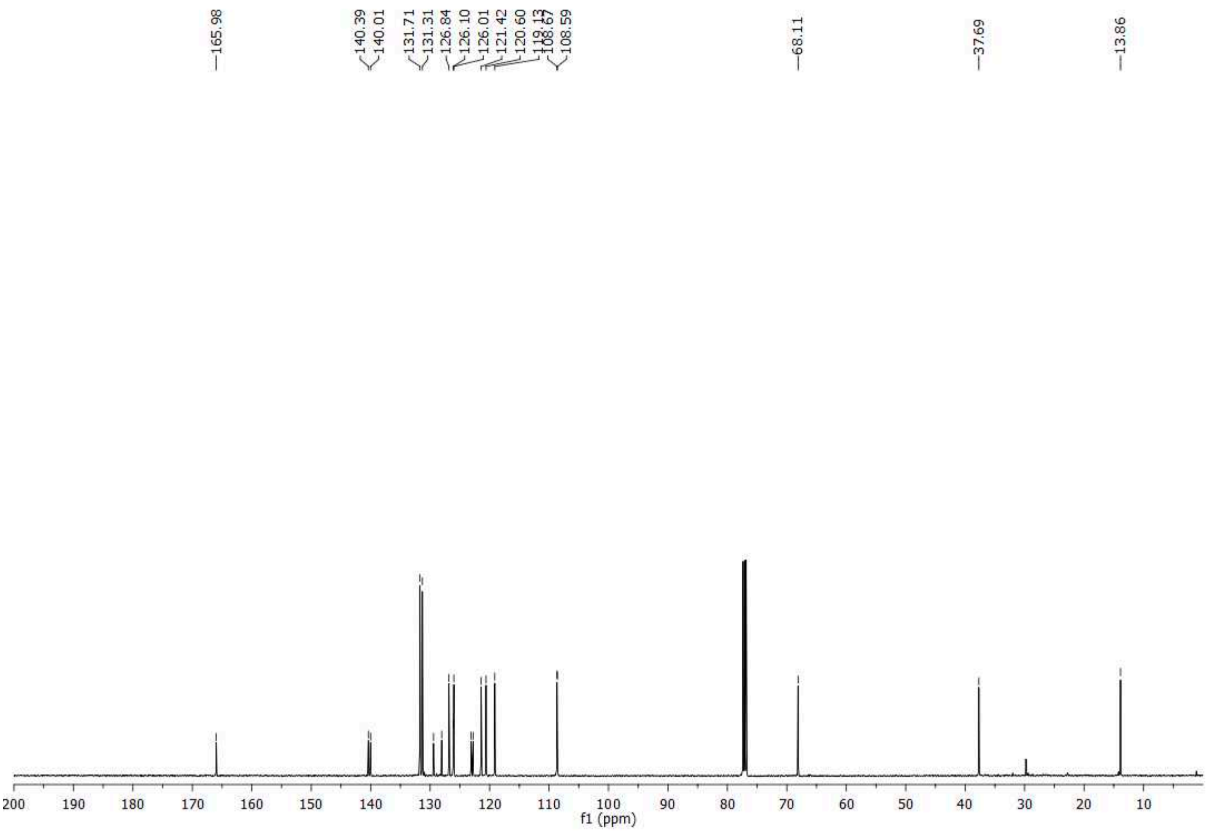

Figure S8: $^{13}C$ NMR spectrum of the compound **5d** (125 MHz, in $CDCl_3$)

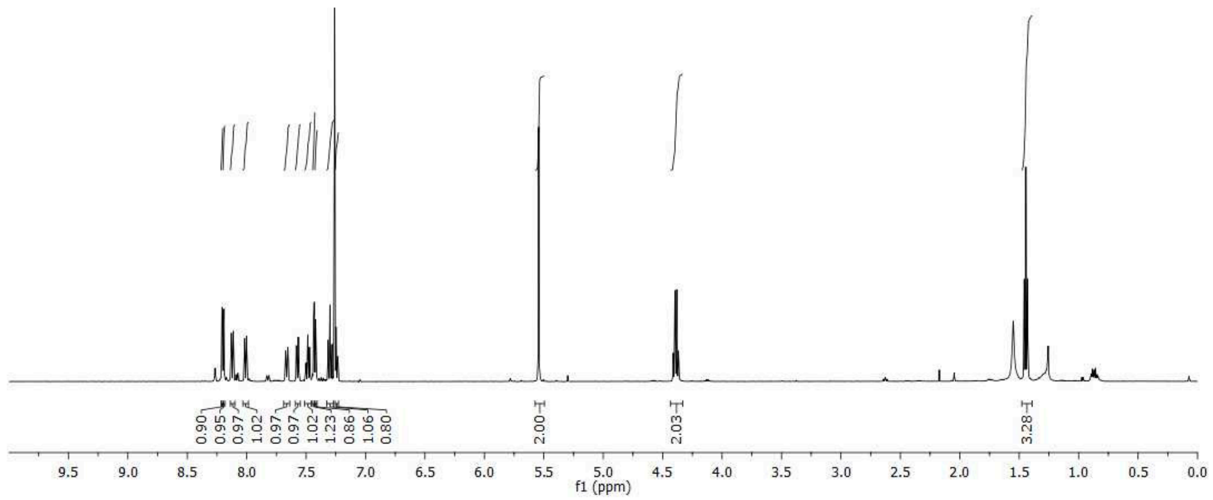


Figure S9: $^{1}$H NMR spectrum of the compound **5e** (500 MHz, in $CDCl_3$)

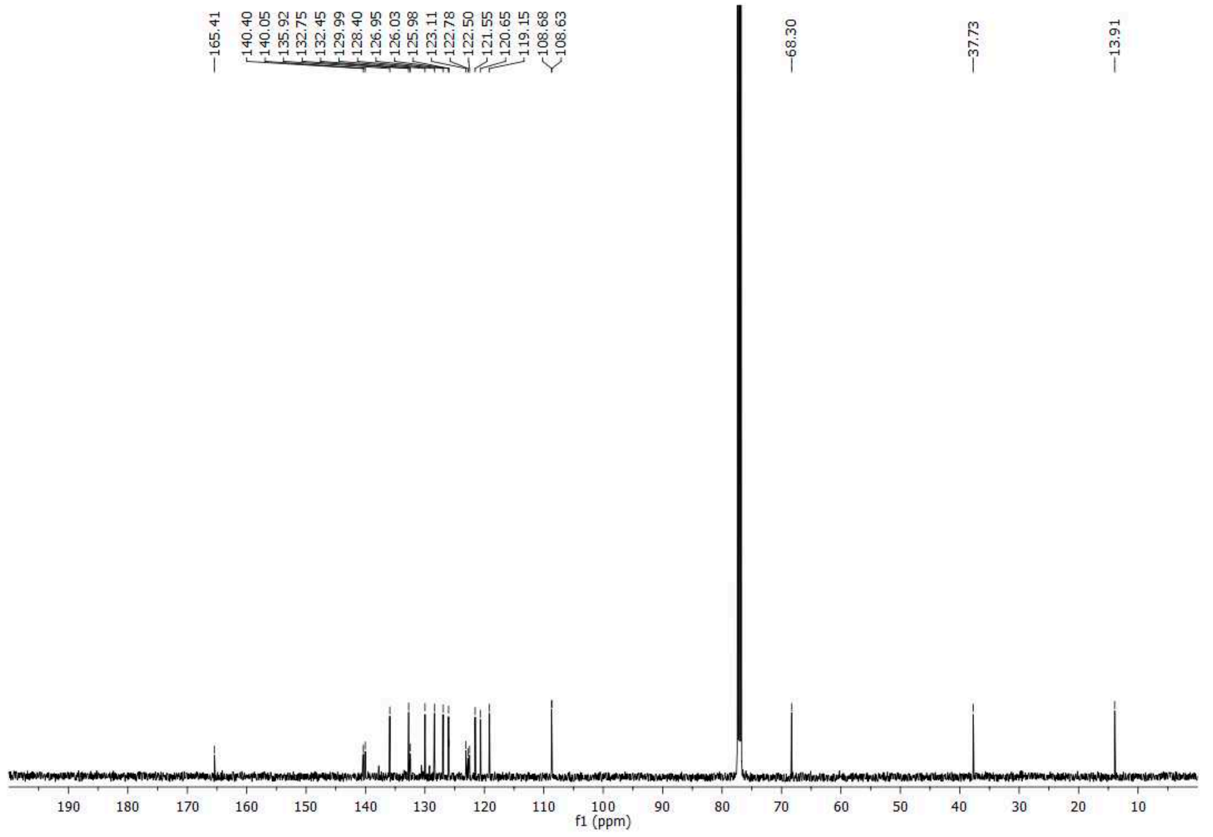


Figure S10: $^{13}$C NMR spectrum of the compound **5e** (125 MHz, in $CDCl_3$)

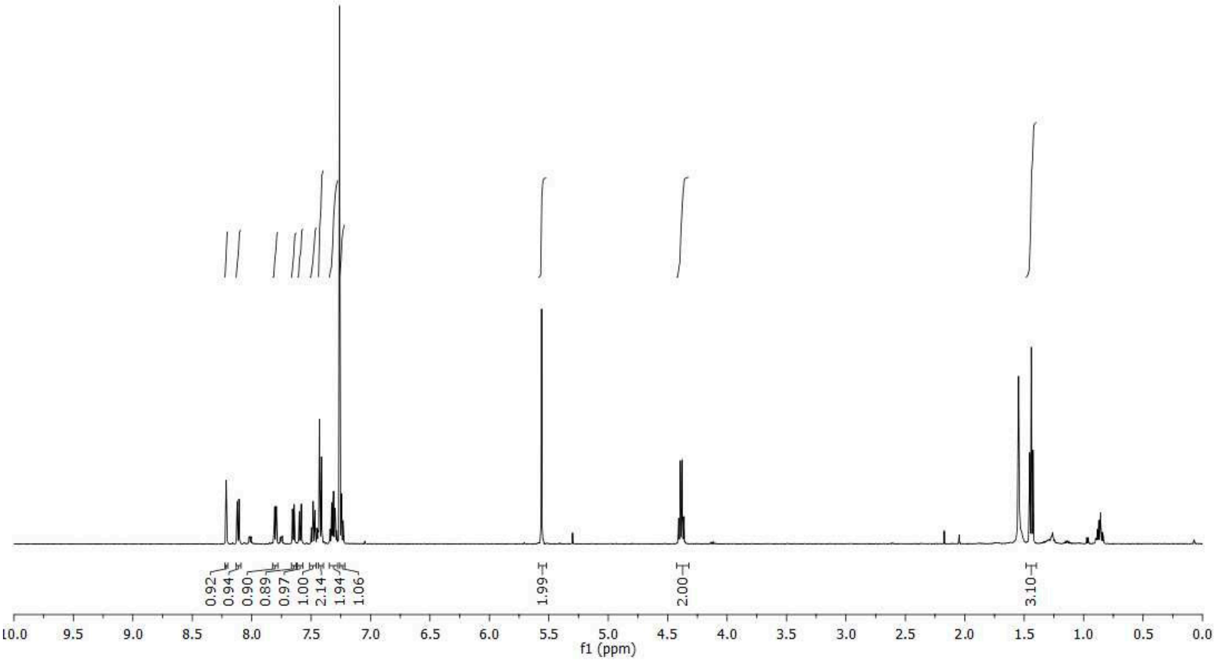


Figure S11: $^{1}$H NMR spectrum of the compound **5f** (500 MHz, in $CDCl_3$)

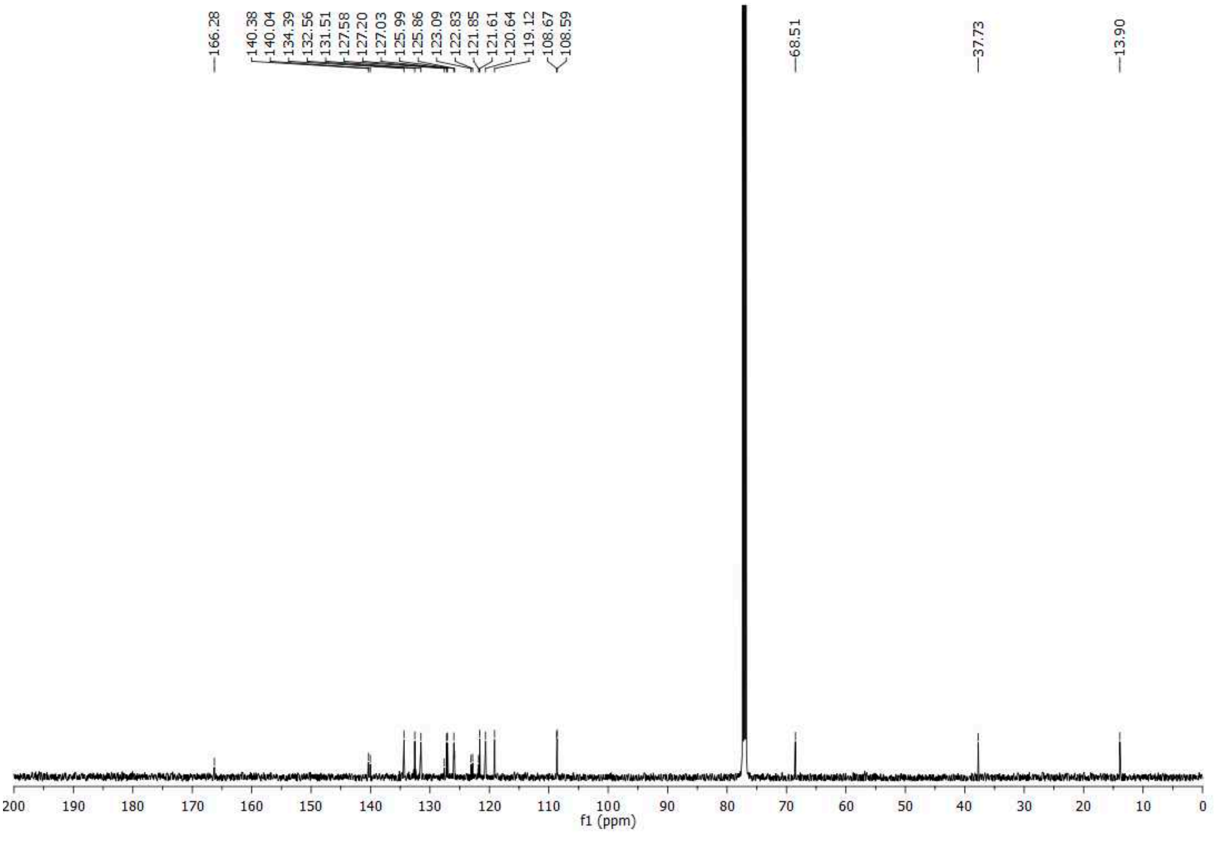


Figure S12: $^{13}$C NMR spectrum of the compound **5f** (125 MHz, in $CDCl_3$)

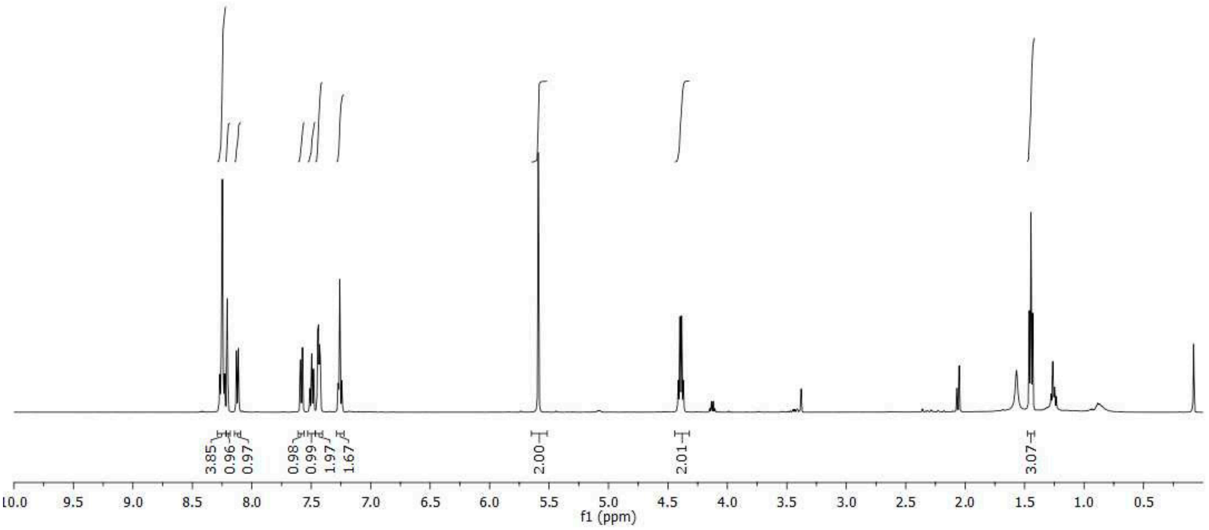

Figure S13: $^{1}H$ NMR spectrum of the compound **5g** (500 MHz, in $CDCl_3$)

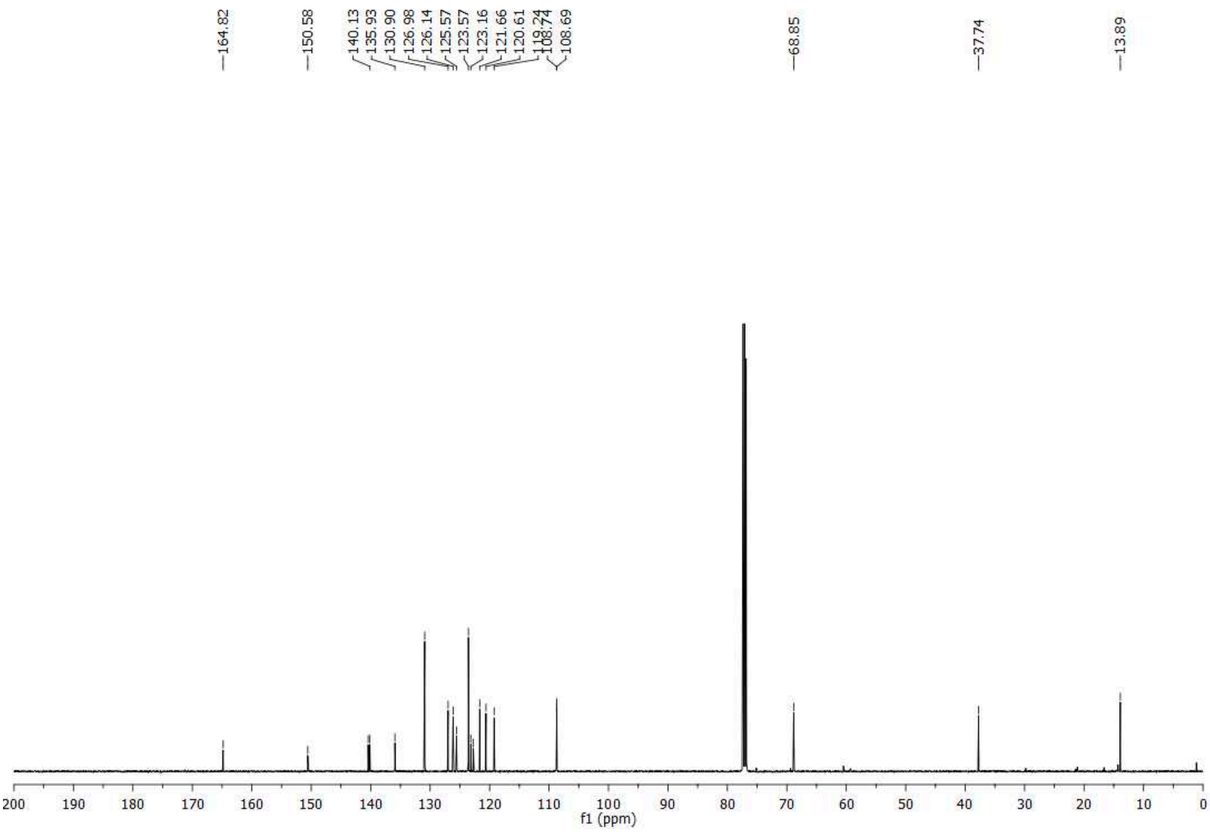

Figure S14: $^{13}C$ NMR spectrum of the compound **5g** (125 MHz, in $CDCl_3$)

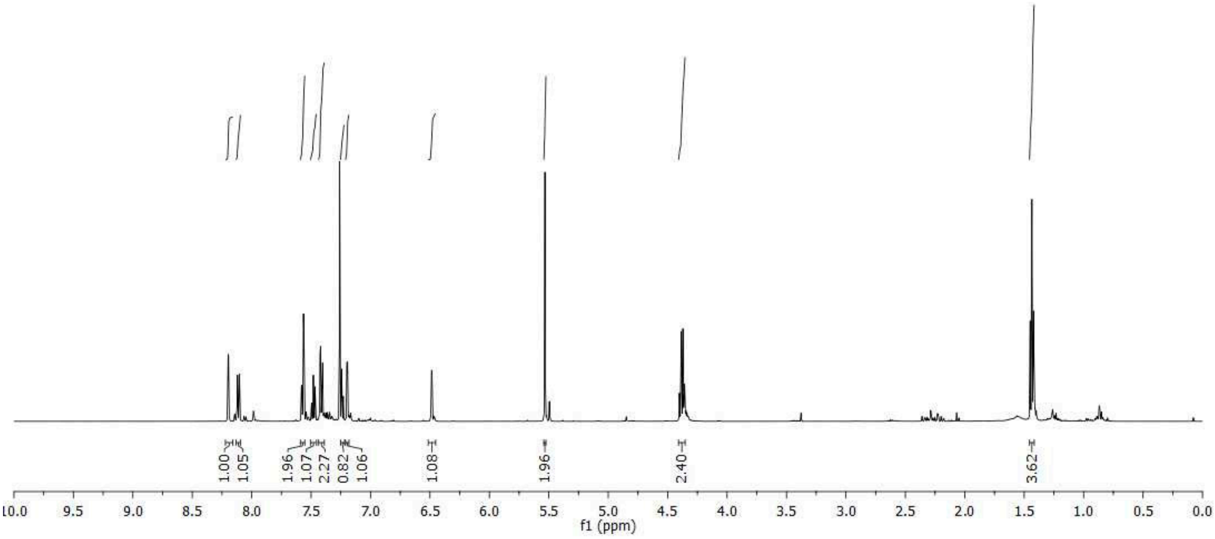


Figure S15: $^{1}$H NMR spectrum of the compound **5h** (500 MHz, in $CDCl_3$)

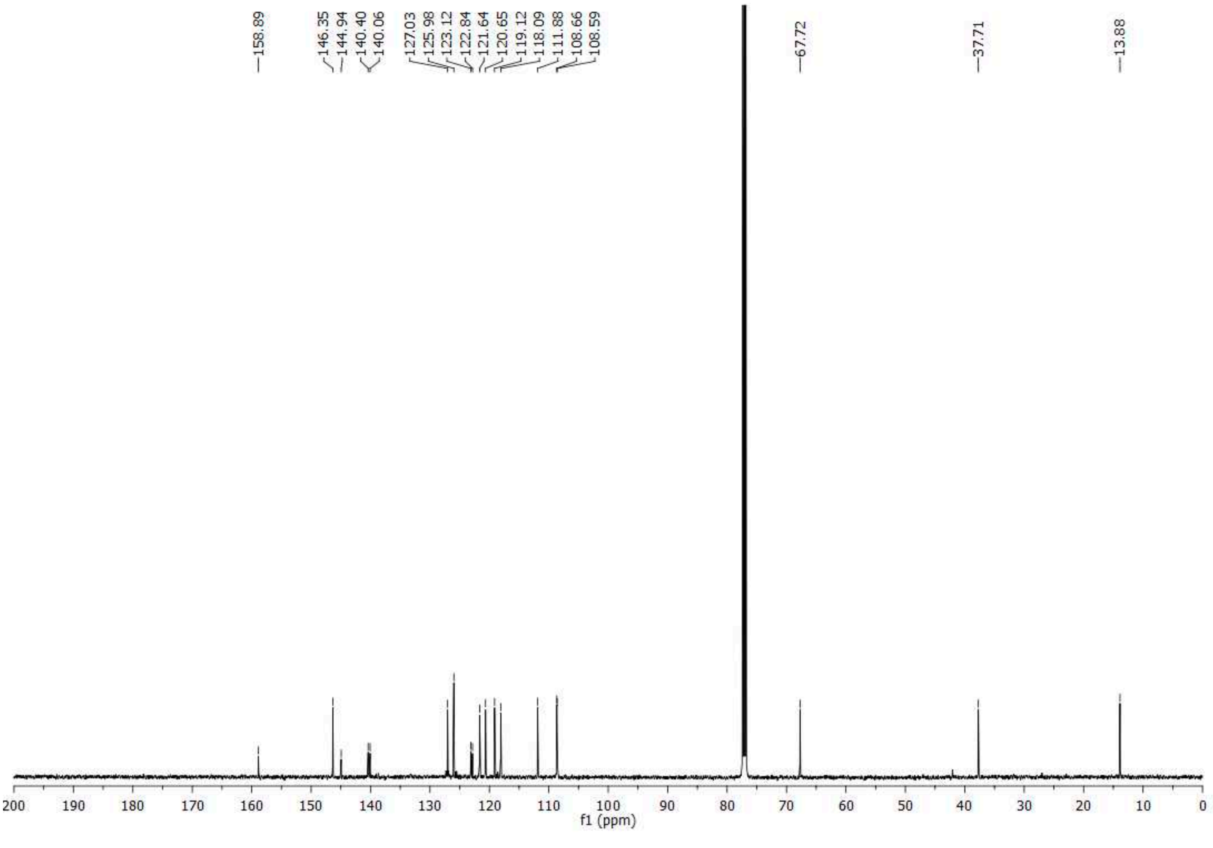


Figure S16: $^{13}$C NMR spectrum of the compound **5h** (125 MHz, in $CDCl_3$)

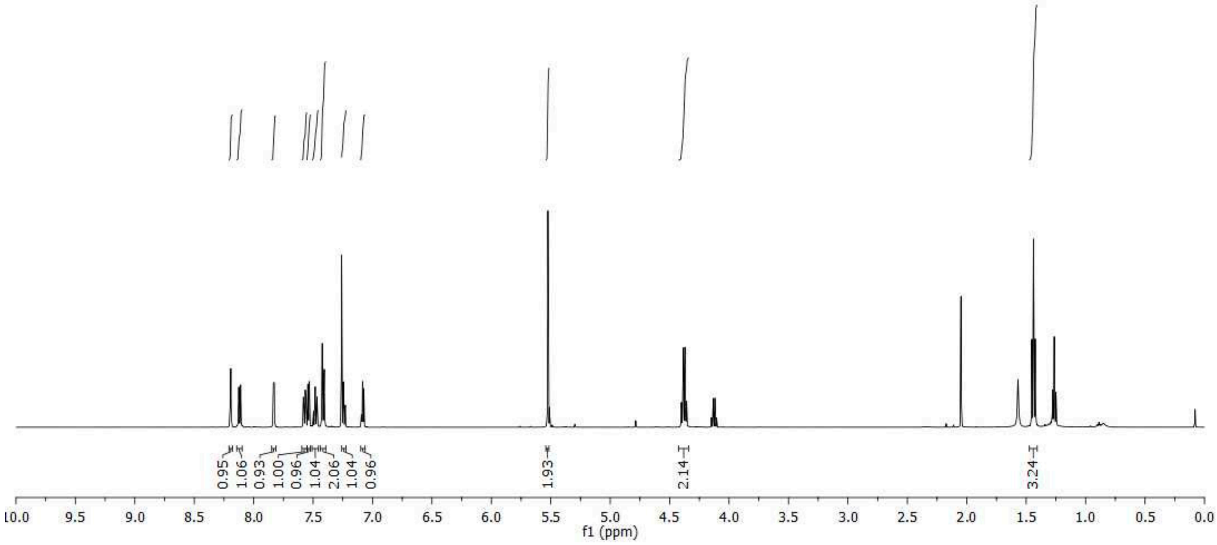


Figure S17: $^{1}H$ NMR spectrum of the compound **5i** (500 MHz, in $CDCl_3$)

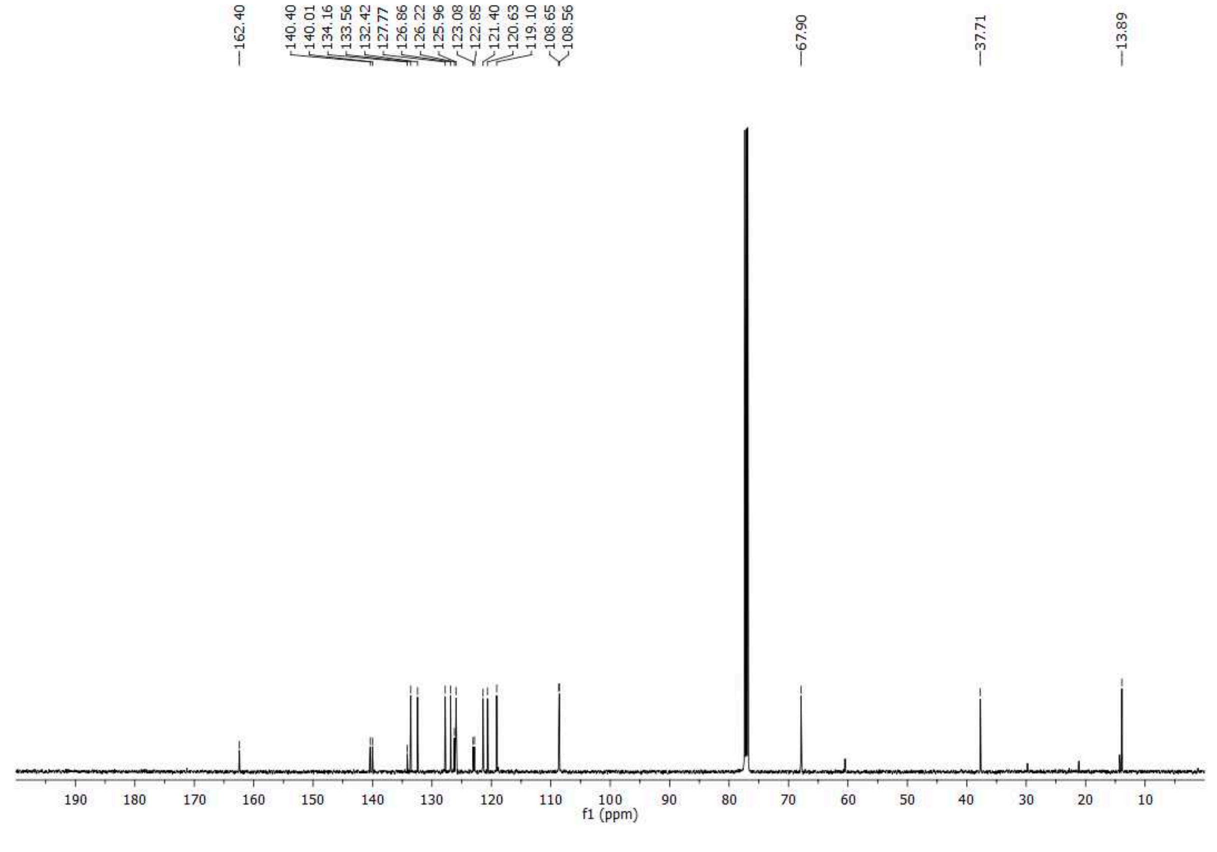


Figure S18: $^{13}C$ NMR spectrum of the compound **5i** (125 MHz, in $CDCl_3$)

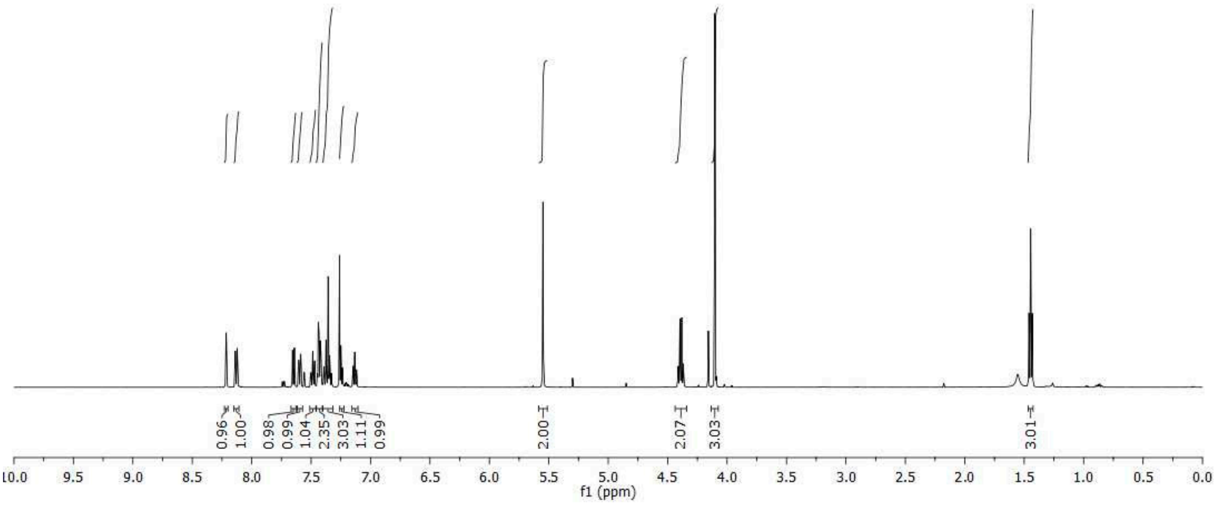

Figure S19: $^{1}H$ NMR spectrum of the compound **5j** (500 MHz, in $CDCl_3$)

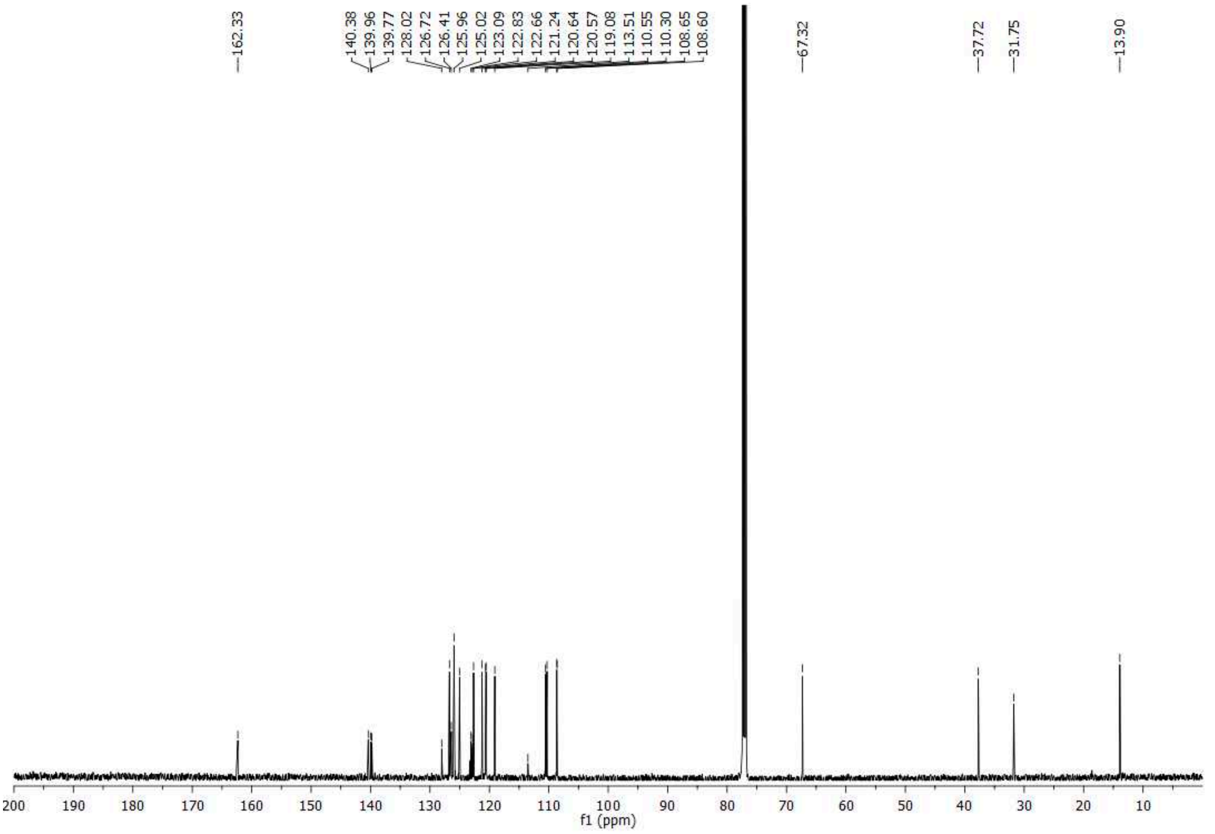

Figure S20: $^{13}C$ NMR spectrum of the compound **5j** (125 MHz, in $CDCl_3$)

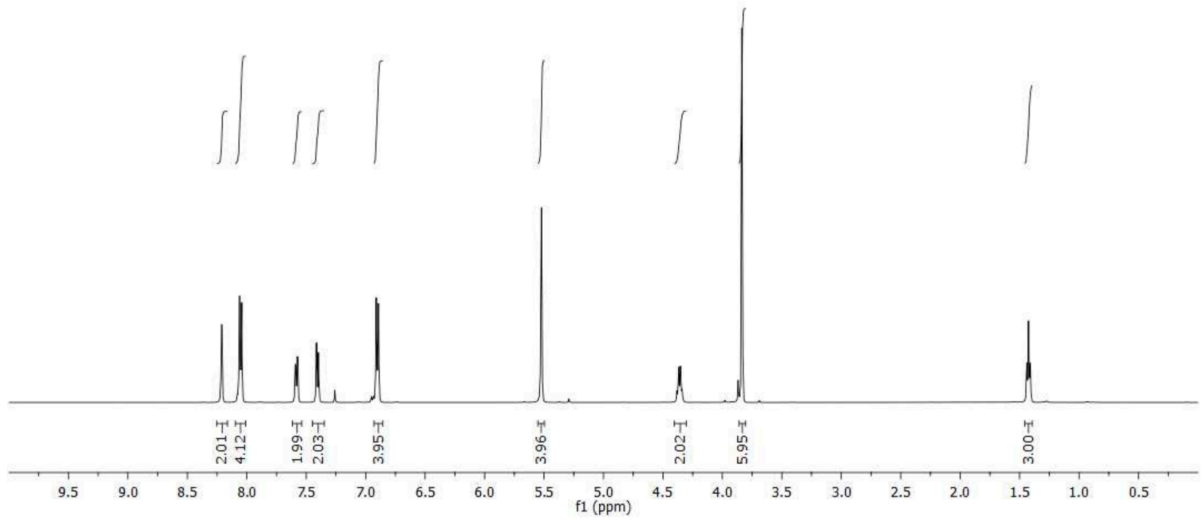


Figure S21: $^{1}$H NMR spectrum of the compound **7a** (500 MHz, in $CDCl_3$)

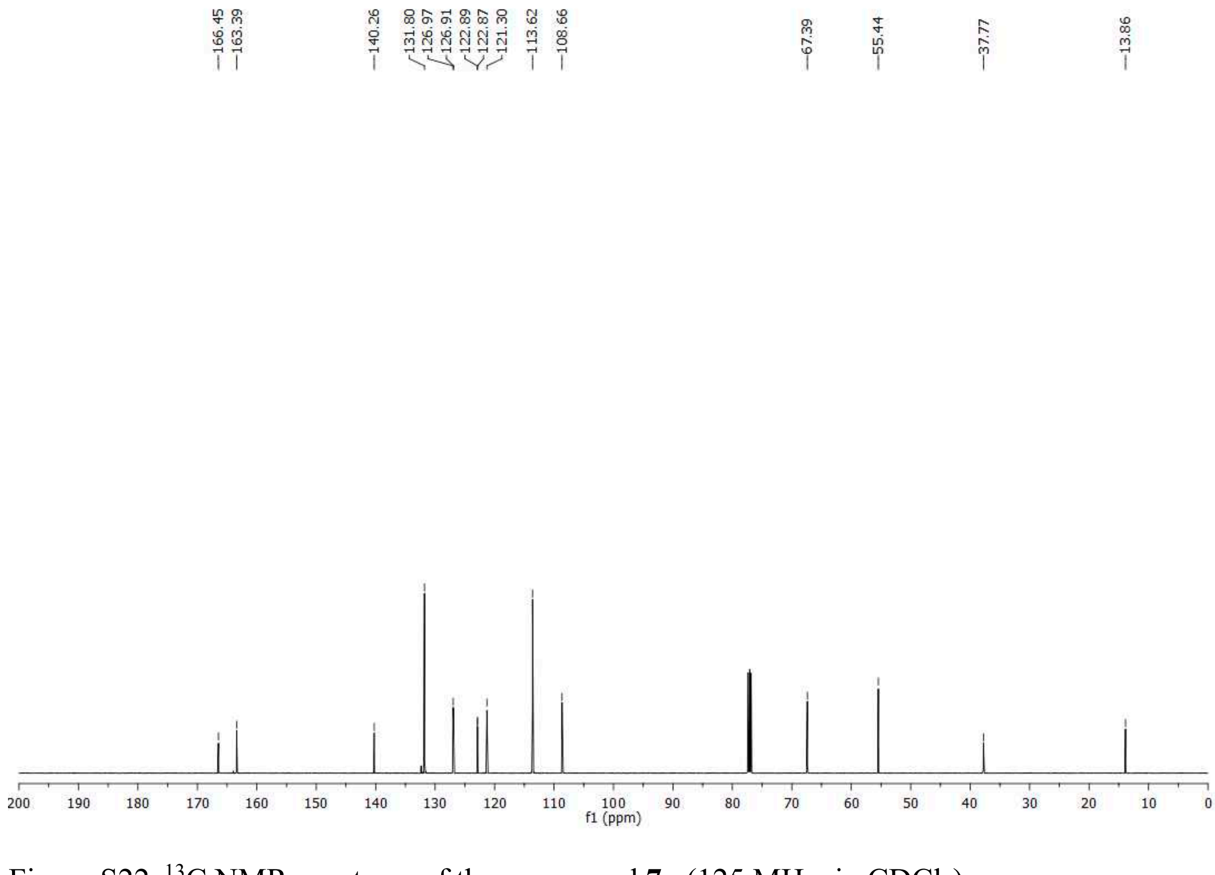


Figure S22: $^{13}$C NMR spectrum of the compound **7a** (125 MHz, in $CDCl_3$)

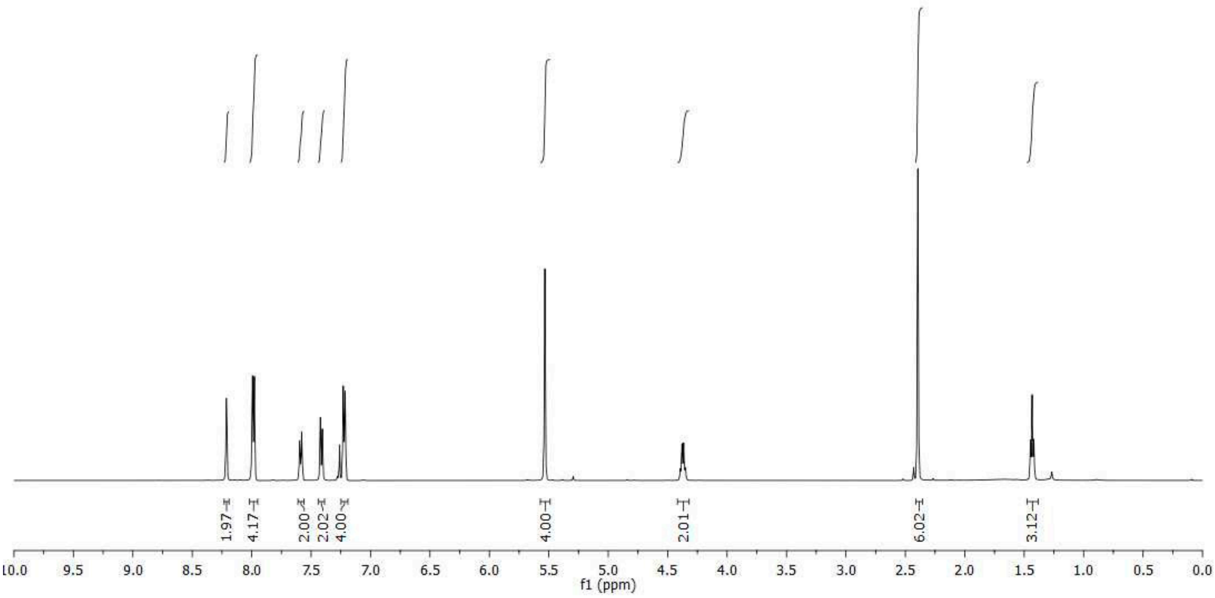


Figure S23: $^{1}$H NMR spectrum of the compound **7b** (500 MHz, in $CDCl_3$)

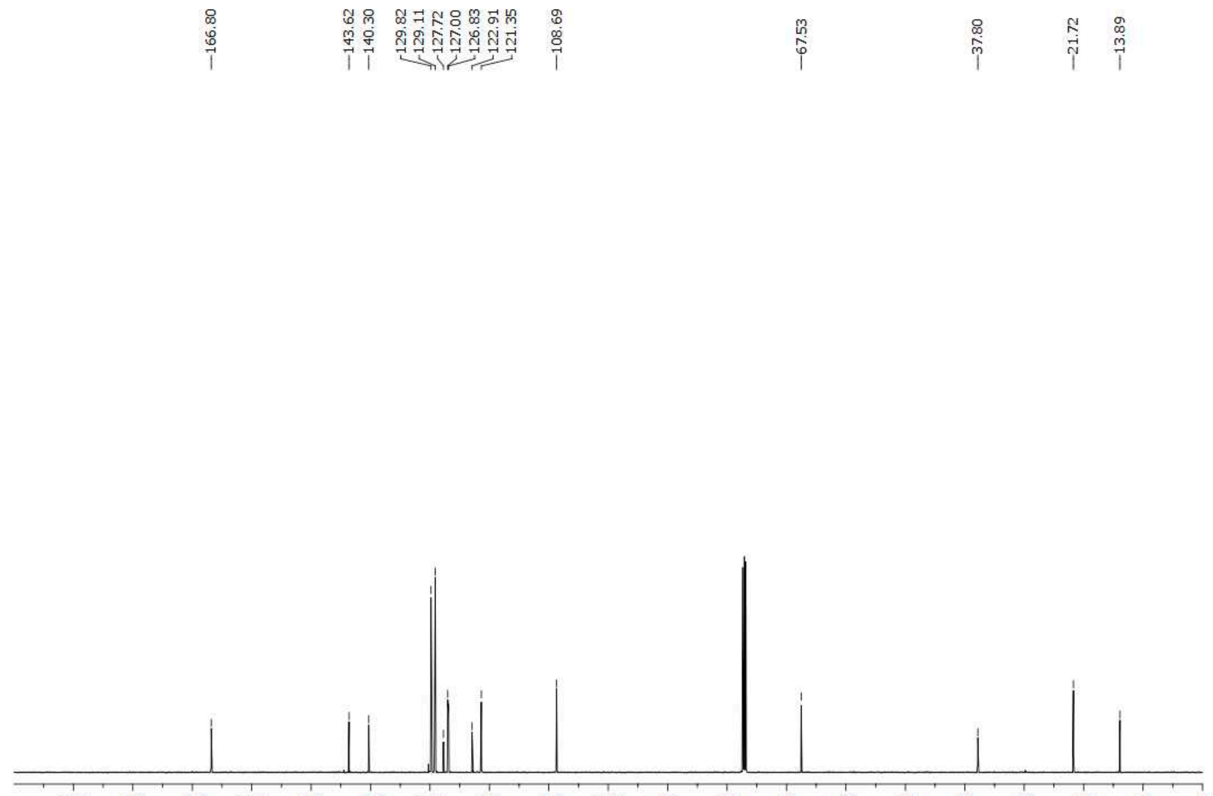


Figure S24: $^{13}$C NMR spectrum of the compound **7b** (125 MHz, in $CDCl_3$)

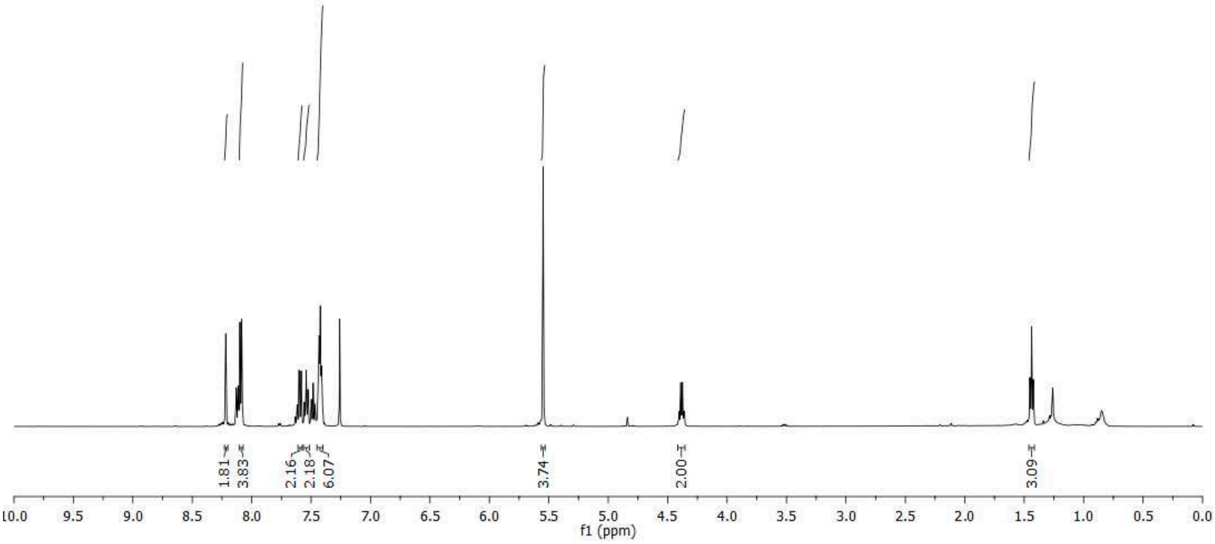


Figure S25: $^{1}$H NMR spectrum of the compound **7c** (500 MHz, in $CDCl_3$)

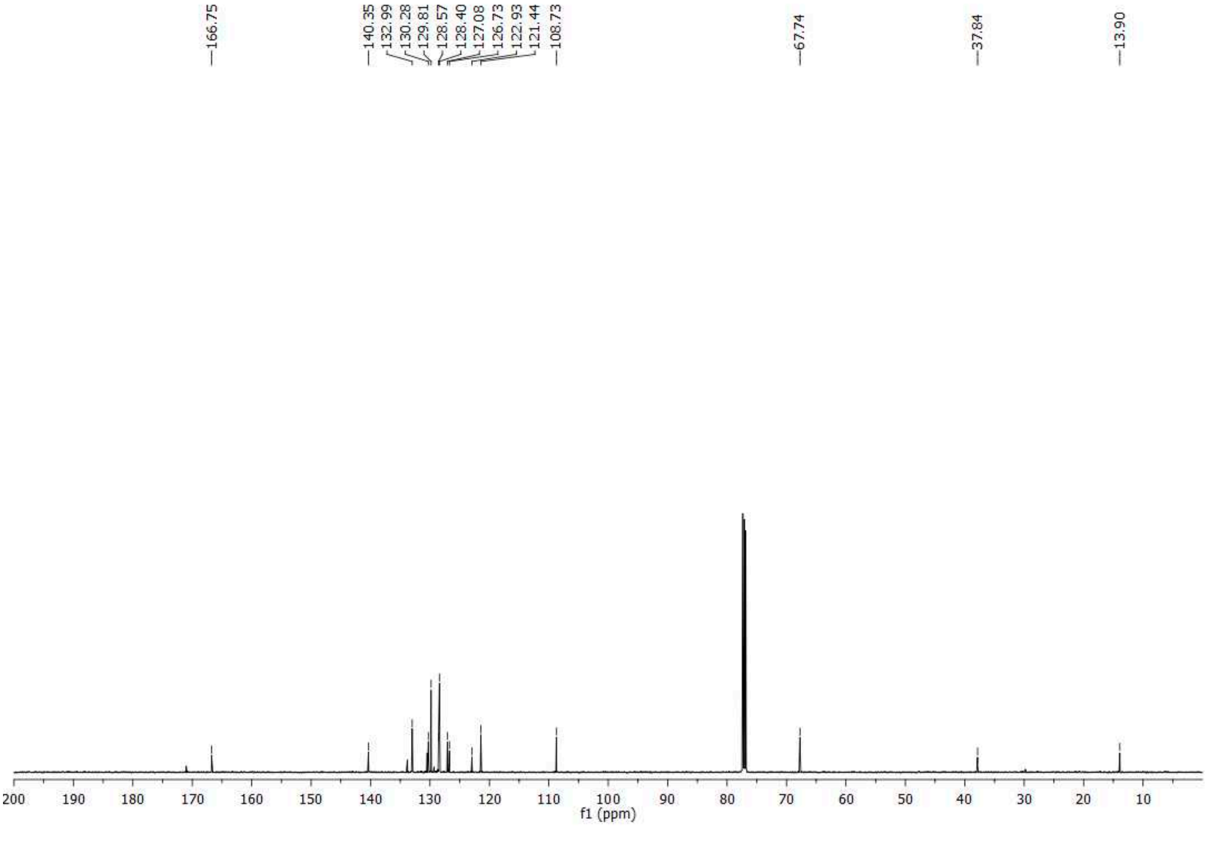


Figure S26: $^{13}$C NMR spectrum of the compound **7c** (125 MHz, in $CDCl_3$)

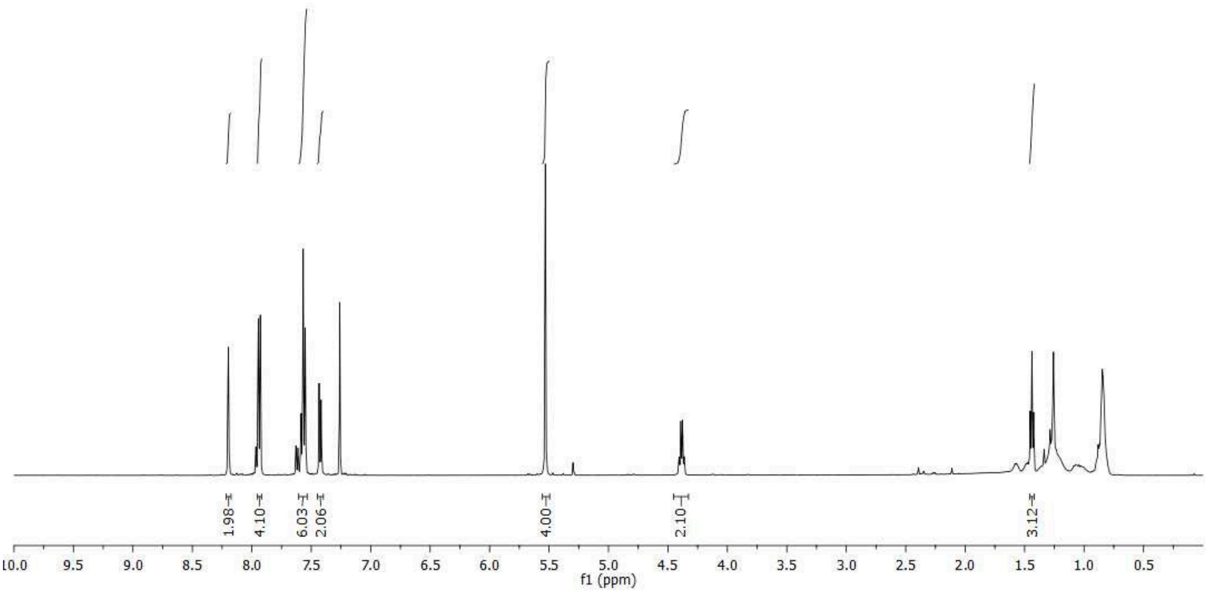


Figure S27: $^{1}H$ NMR spectrum of the compound **7d** (500 MHz, in $CDCl_3$)

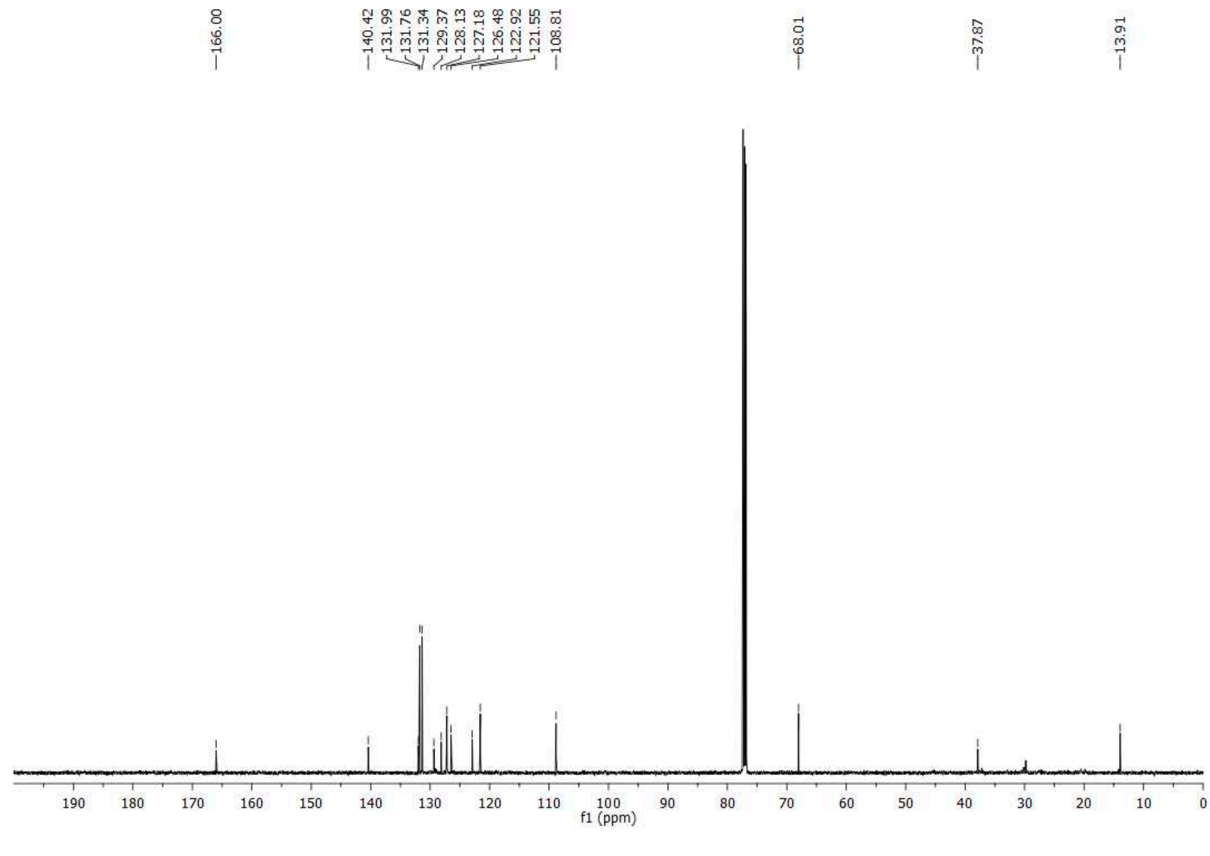


Figure S28: $^{13}C$ NMR spectrum of the compound **7d** (125 MHz, in $CDCl_3$)

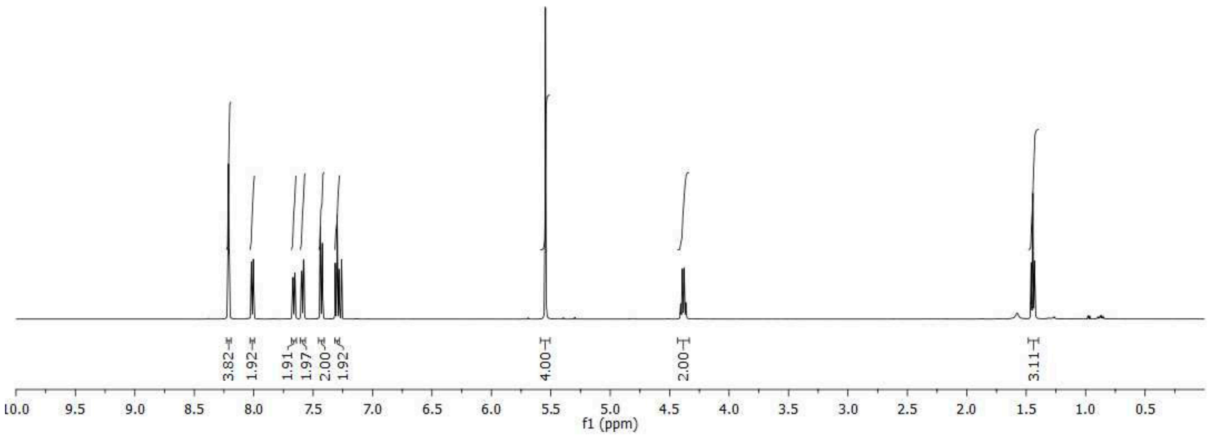


Figure S29: $^{1}$H NMR spectrum of the compound **7e** (500 MHz, in $CDCl_3$)

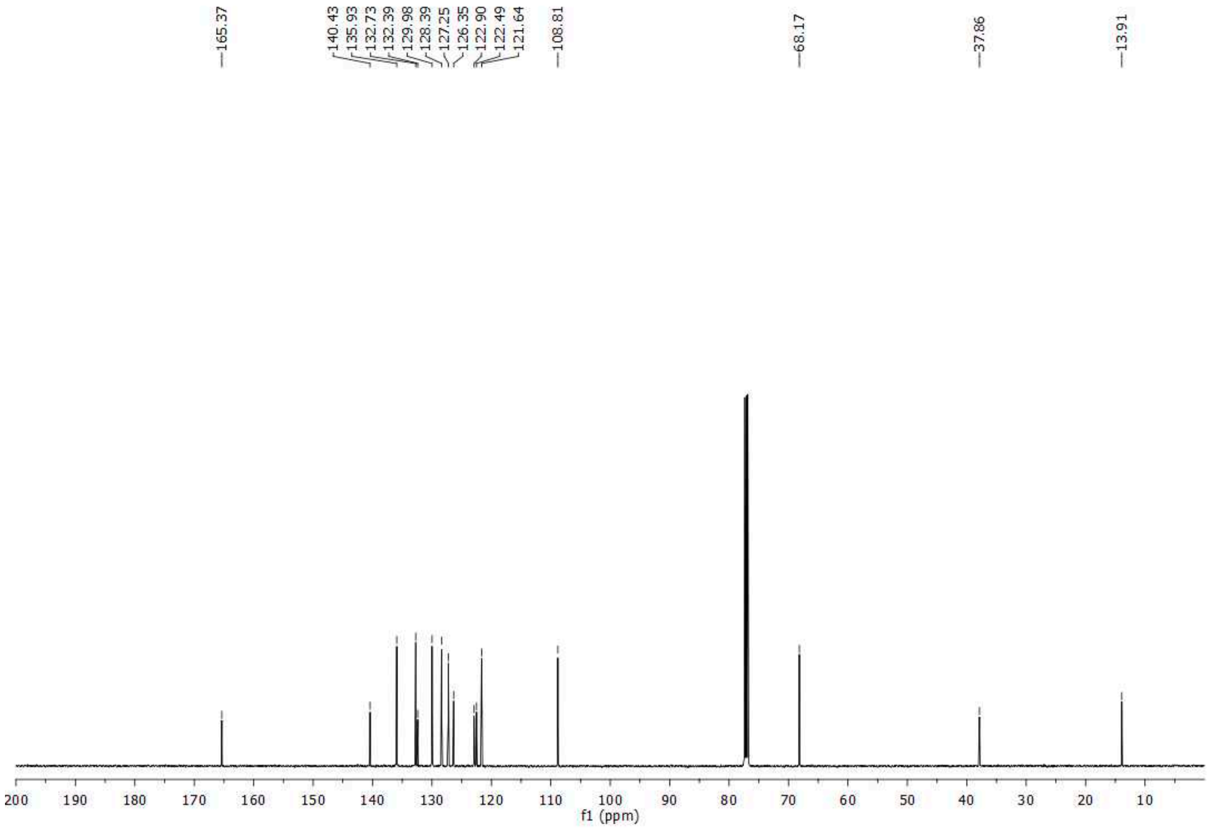


Figure S30: $^{13}$C NMR spectrum of the compound **7e** (125 MHz, in $CDCl_3$)

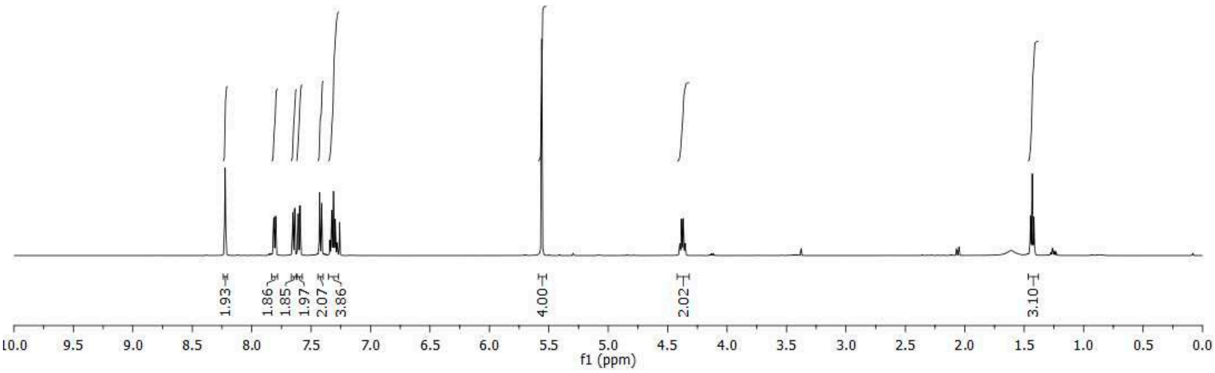


Figure S31: $^{1}$H NMR spectrum of the compound **7f** (500 MHz, in $CDCl_3$)

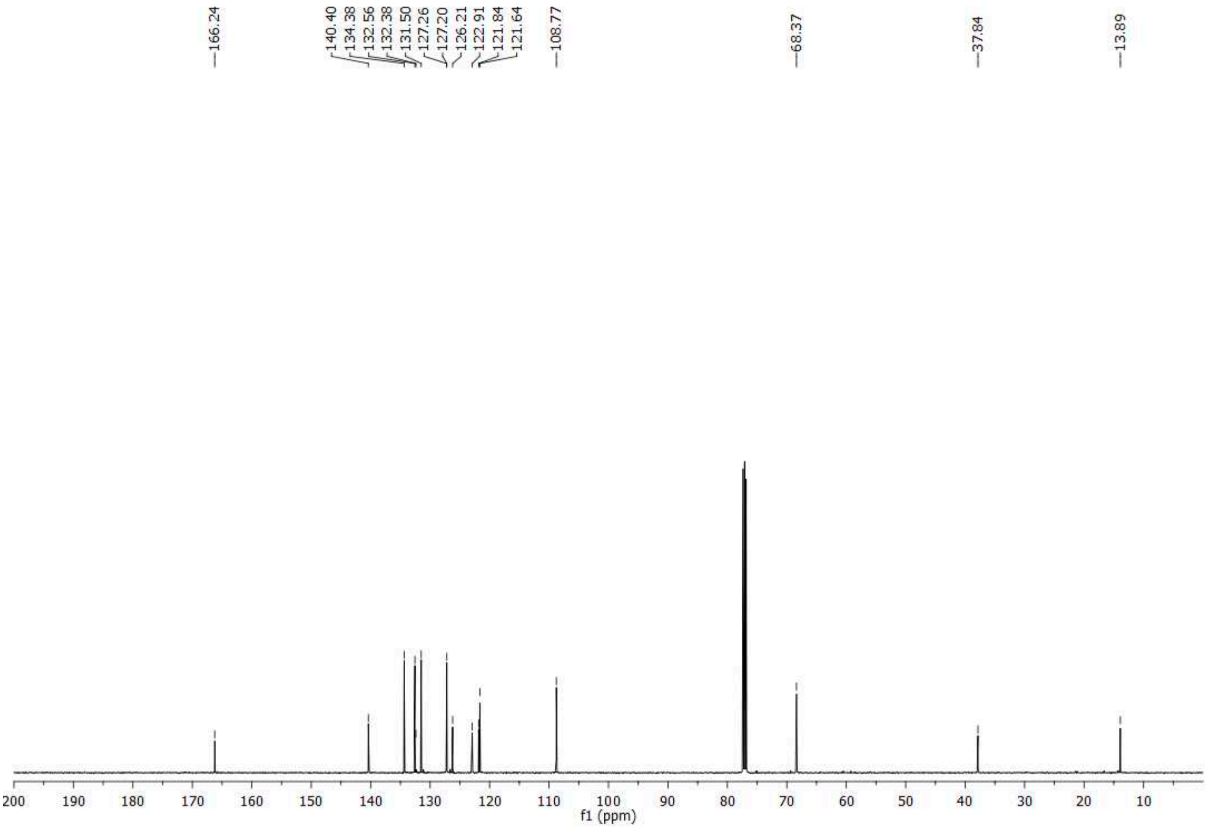


Figure S32: $^{13}$C NMR spectrum of the compound **7f** (125 MHz, in $CDCl_3$)

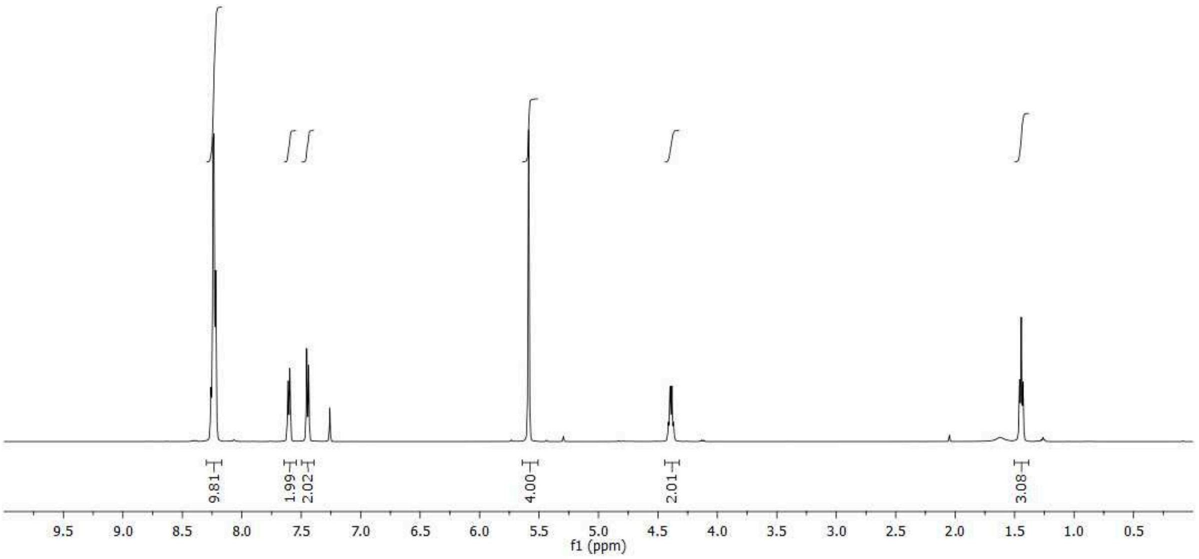


Figure S33: $^{1}$H NMR spectrum of the compound **7g** (500 MHz, in $CDCl_3$)

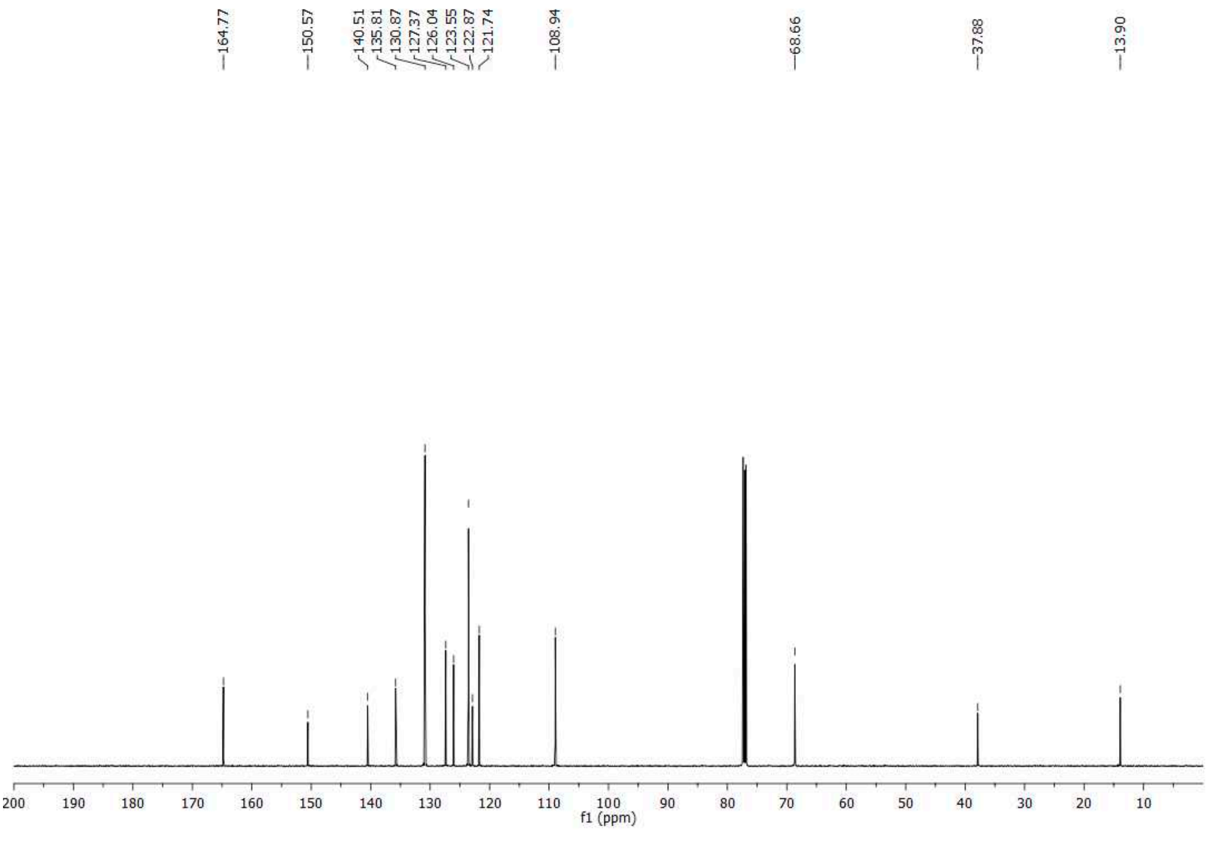


Figure S34: $^{13}$C NMR spectrum of the compound **7g** (125 MHz, in $CDCl_3$)

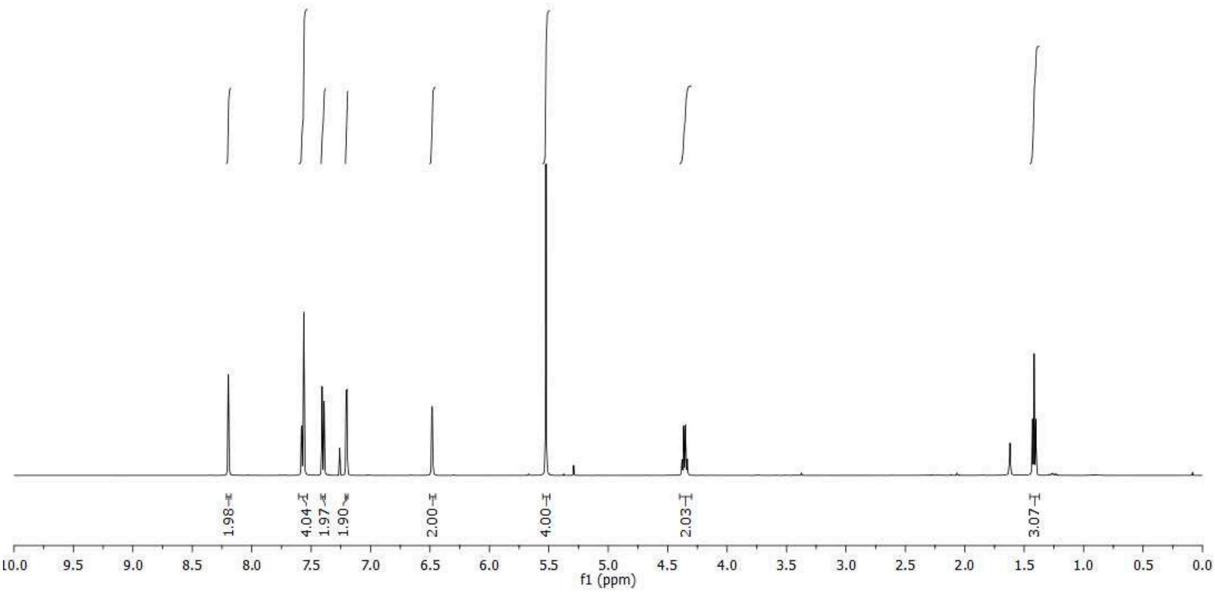


Figure S35: $^{1}$H NMR spectrum of the compound **7h** (500 MHz, in $CDCl_3$)

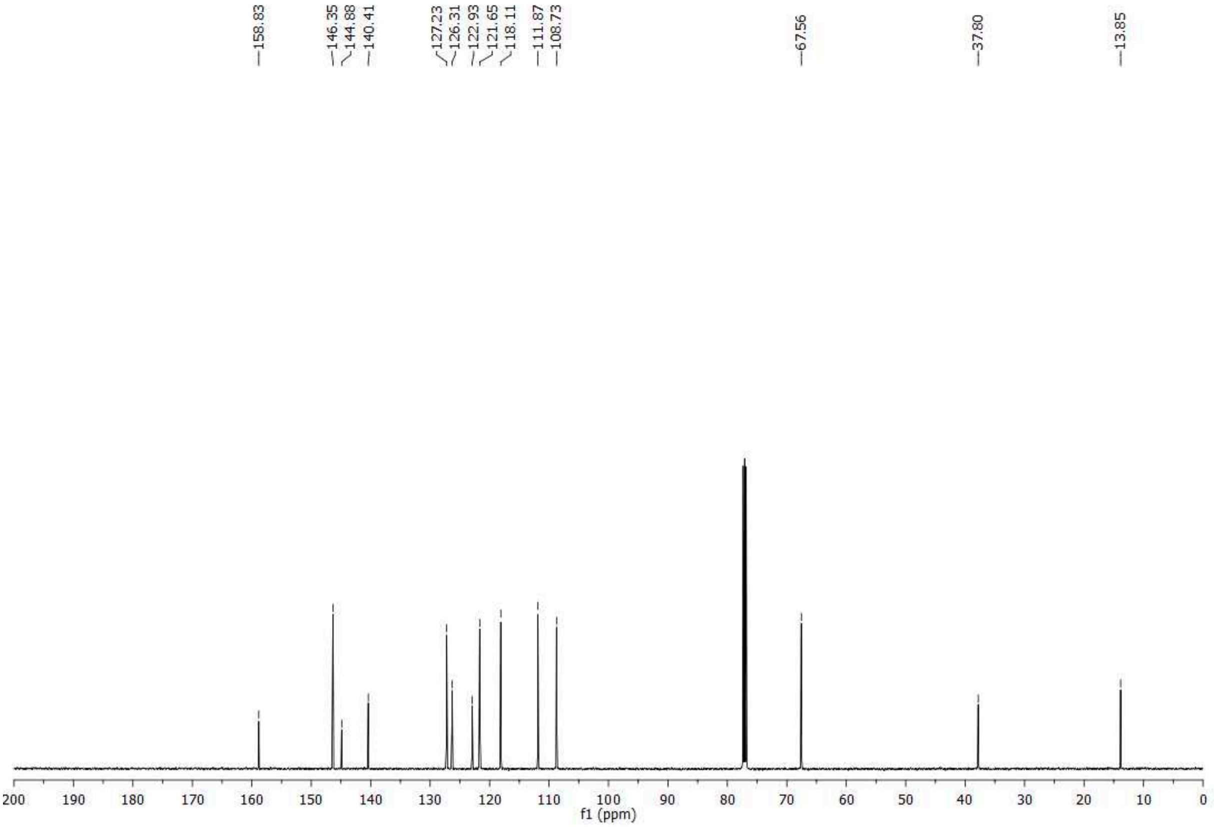


Figure S36: $^{13}$C NMR spectrum of the compound **7h** (125 MHz, in $CDCl_3$)

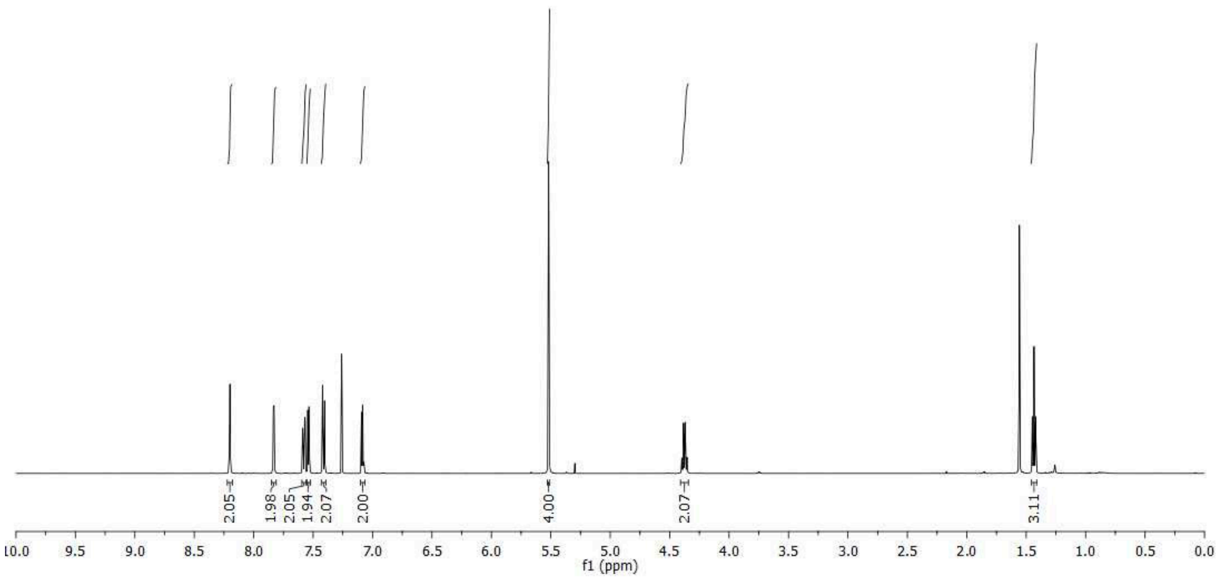


Figure S37: $^{1}$H NMR spectrum of the compound **7i** (500 MHz, in $CDCl_3$)

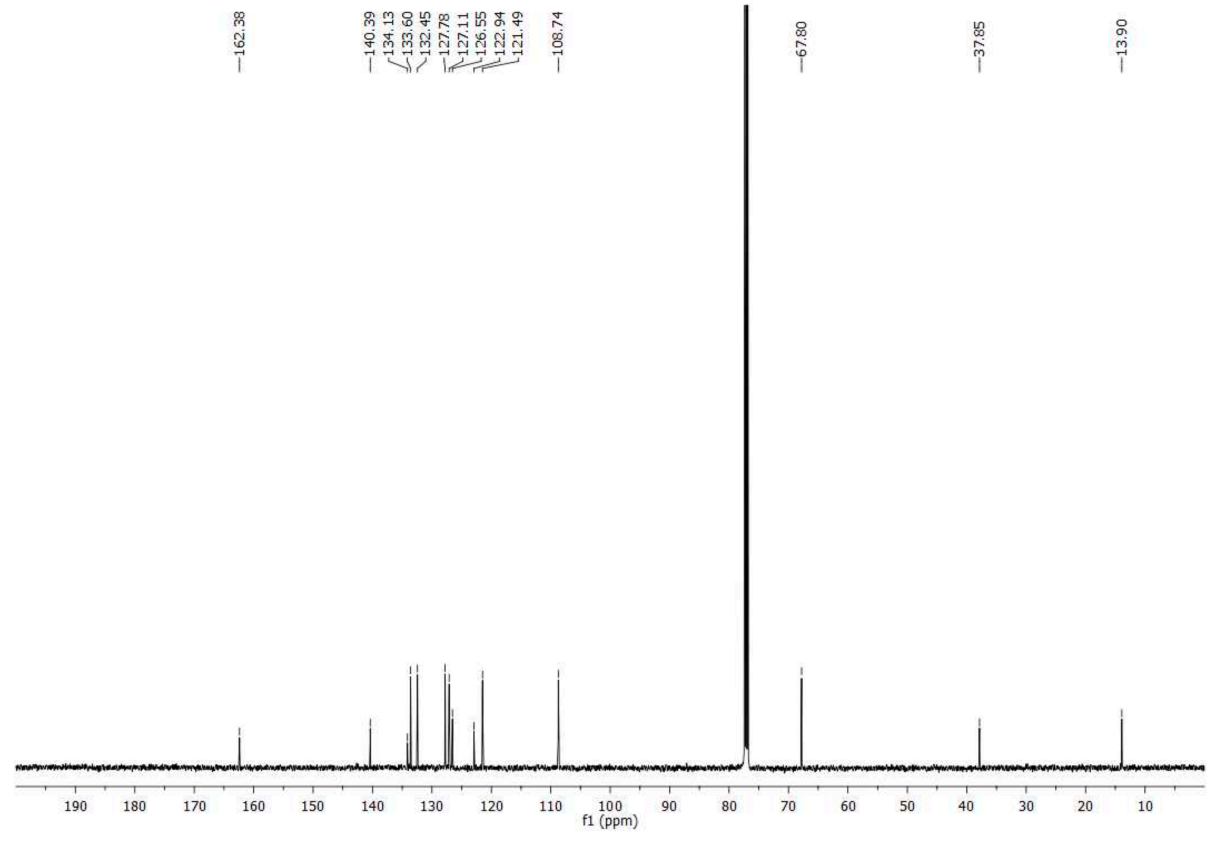


Figure S38: $^{13}$C NMR spectrum of the compound **7i** (125 MHz, in $CDCl_3$)

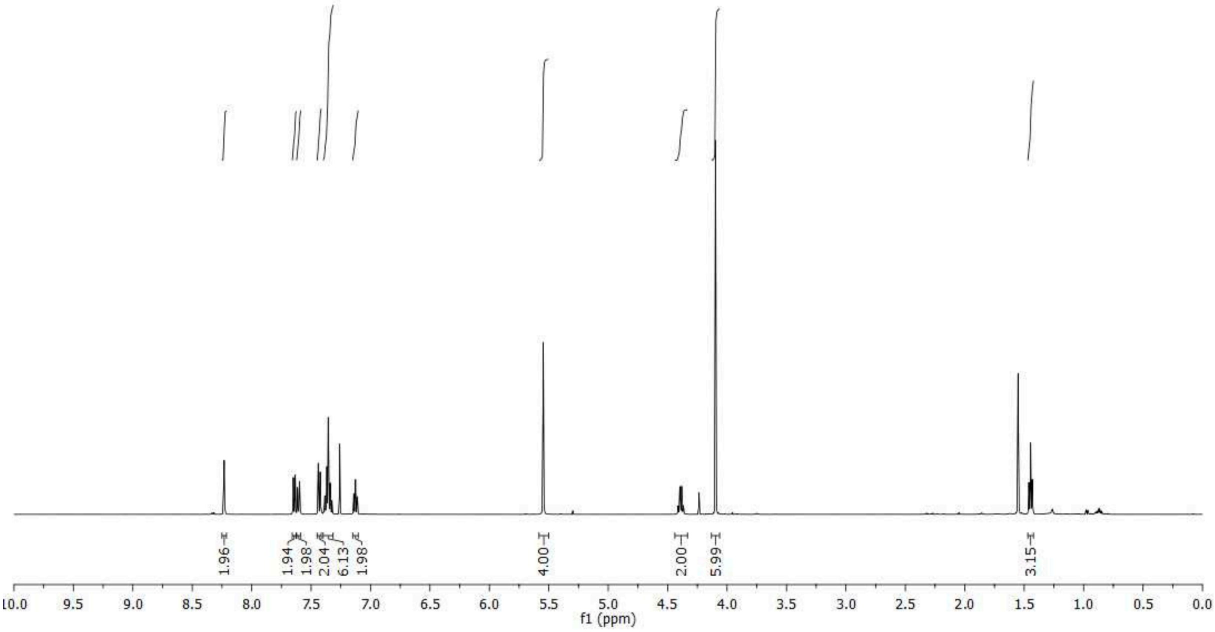

Figure S39: $^{1}H$ NMR spectrum of the compound **7j** (500 MHz, in $CDCl_3$)

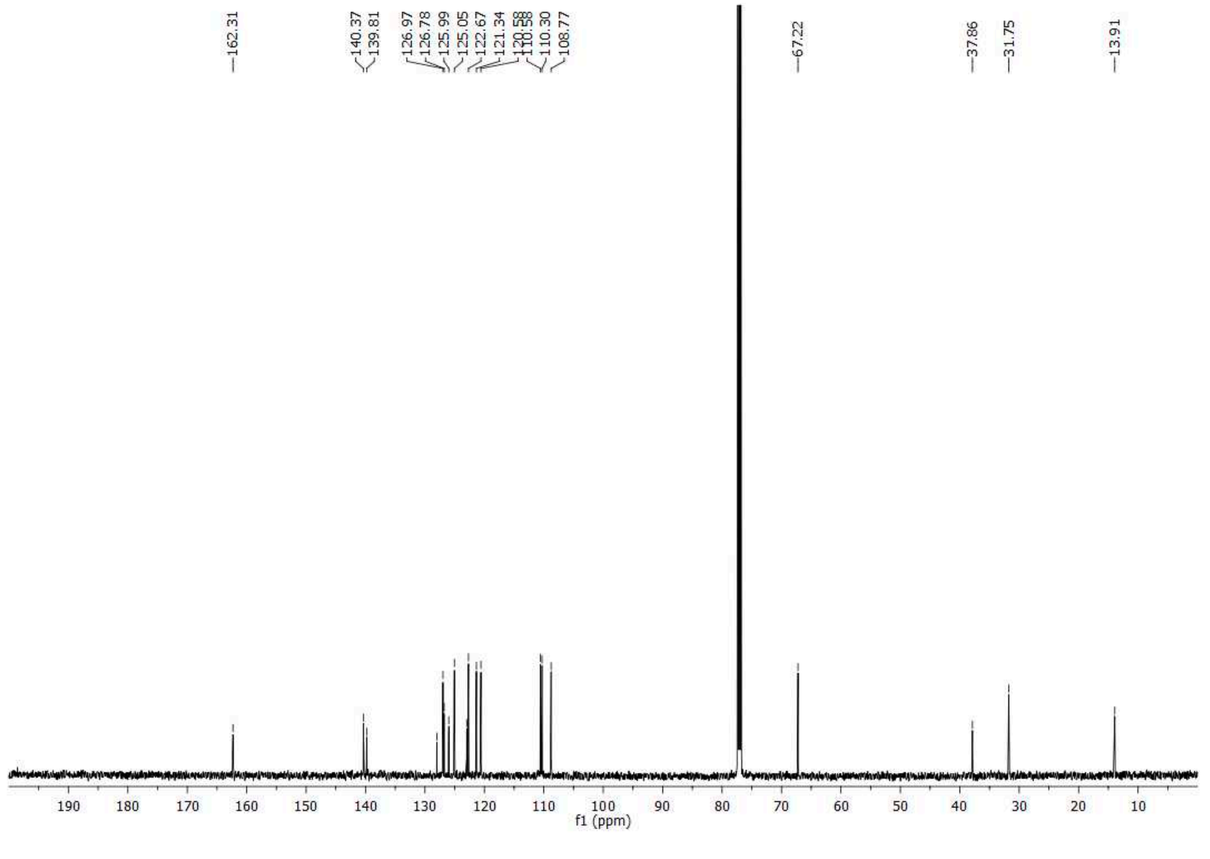

Figure S40: $^{13}C$ NMR spectrum of the compound **7j** (125 MHz, in $CDCl_3$)

## 2. X-ray crystallographic data and structure refinement

**Table S1.** Crystal data and structure refinement details for compound **5j**.

| | |
|---|---|
| Identification code | 25gtu158_MAS_RS_A1_0m |
| Empirical formula | $C_{25}H_{22}N_2O_2$ |
| Formula weight | 382.44 |
| Temperature/K | 273.15 |
| Crystal system | orthorhombic |
| Space group | Pbca |
| a/Å | 15.778(6) |
| b/Å | 8.619(3) |
| c/Å | 29.049(10) |
| α/° | 90 |
| β/° | 90 |
| γ/° | 90 |
| Volume/Å$^3$ | 3950(2) |
| Z | 8 |
| $\rho_{calc}$g/cm$^3$ | 1.286 |
| μ/mm$^{-1}$ | 0.082 |
| F(000) | 1616.0 |
| Crystal size/mm$^3$ | 0.302 × 0.16 × 0.138 |
| Radiation | MoKα (λ = 0.71073) |
| 2Θ range for data collection/° | 2.804 to 50 |
| Index ranges | -18 ≤ h ≤ 18, -10 ≤ k ≤ 10, -34 ≤ l ≤ 34 |
| Reflections collected | 39520 |
| Independent reflections | 3480 [$R_{int}$ = 0.1618, $R_{sigma}$ = 0.1107] |
| Data/restraints/parameters | 3480/0/265 |
| Goodness-of-fit on F$^2$ | 1.000 |
| Final R indexes [I>=2σ (I)] | $R_1$ = 0.0593, w$R_2$ = 0.1410 |
| Final R indexes [all data] | $R_1$ = 0.1286, w$R_2$ = 0.1664 |
| Largest diff. peak/hole / e Å$^{-3}$ | 0.18/-0.19 |

## 3.Material and Methods for Docking study

The PDB files corresponding to the docked complexes of the target protein with compound 7g, Acarbose, and compound 5g have been provided as Supplementary Files S1–S5.

- Supplementary File S1. Docked complex of α-amylase with compound 7g (PDB format).
- Supplementary File S2. Docked complex of α-glucosidase with compound 7g (PDB format).
- Supplementary File S3. Docked complex of α- amylase with Acarbose (PDB format).
- Supplementary File S4. Docked complex of α-glucosidase with Acarbose (PDB format).
- Supplementary File S5. Docked complex of α-amylase with compound 5g (PDB format).
